# Analysis of complex drugs by comprehensive two-dimensional gas chromatography and high-resolution mass spectrometry: detailed chemical description of the active pharmaceutical ingredient sodium bituminosulfonate and its process intermediates

**DOI:** 10.1007/s00216-022-04393-w

**Published:** 2022-11-19

**Authors:** Lukas Schwalb, Ole Tiemann, Uwe Käfer, Thomas Gröger, Christopher Paul Rüger, Guido Gayko, Ralf Zimmermann

**Affiliations:** 1 Joint Mass Spectrometry Centre (JMSC), Chair of Analytical Chemistry, University of Rostock, Rostock, Germany; 2 Joint Mass Spectrometry Centre (JMSC), Cooperation Group “Comprehensive Molecular Analytics” (CMA), Helmholtz Zentrum München GmbH, German Research Center for Environmental Health, Neuherberg, Germany; 3 Now at: Leibniz-Institute of Tropospheric Research (TROPOS), Leipzig, Germany; 4 Ichthyol-Gesellschaft, Cordes, Hermanni & Co. (GmbH & Co.) KG, Hamburg, Germany

**Keywords:** GC × GC, HR-MS, Classification, Non-biological complex drugs, Complex drugs, Sodium bituminosulfonate

## Abstract

The European pharmacopeia provides analytical methods for the chemical characterization of active pharmaceutical ingredients (APIs). However, the complexity of some APIs exceeds the limitations of the currently prevailing physicochemical methods. Sodium bituminosulfonate (SBS) is described by the collection of key parameters of generalizing criteria such as dry matter, sulfur and sodium content, and neutrality, but techniques to unravel the complexity on a molecular level are lacking. We present a study based on online derivatization with tetramethylammonium hydroxide in combination with comprehensive two-dimensional gas chromatography coupled to an electron ionization high-resolution time-of-flight mass spectrometer (GC × GC-HR-ToF–MS) for the chemical description of SBS as well as its process intermediates. The application of GC × GC allowed the comprehensive description of the chemical components in the API and the process intermediates for the first time. Furthermore, it was possible to classify peaks regarding their elemental and structural composition based on accurate mass information, elution behavior, and mass fragmentation pattern. This work demonstrates not only the general applicability, advantages but also limitations of GC × GC for the characterization of APIs for complex drugs.

**Graphical Abstract:**

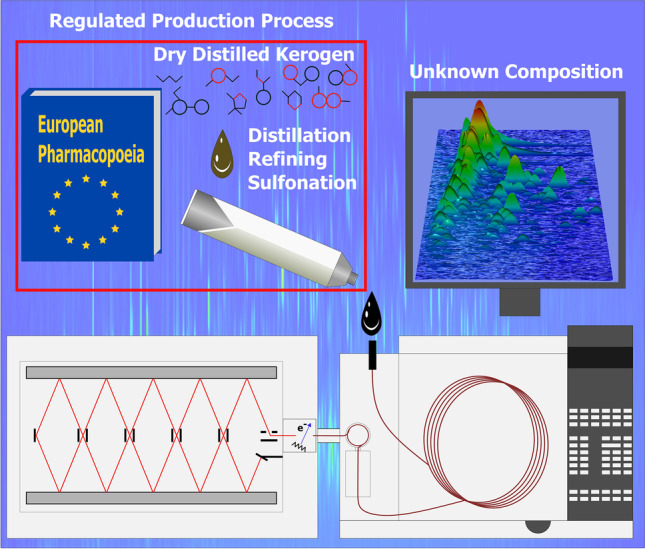

**Supplementary Information:**

The online version contains supplementary material available at 10.1007/s00216-022-04393-w.

## Introduction

The chemical analysis and characterization of drugs is a typical task of the certification and release process in the pharmaceutical industry. Some active pharmaceutical ingredients (APIs), however, are characterized by a complexity that cannot be unraveled by standard methods described in the European or Untied States Pharmacopeia. In recent years, the analysis of the pharmaceutical group of non-biological complex drugs (NBCDs) is coming into the focus of regulatory affairs [1, 2]. NBCDs comprise APIs of non-biological origin that do not have a uniform (homo-) molecular structure [3]. Due to their complexity, it is impossible to isolate single components and to characterize the drugs by common physicochemical analytical methods found in the Pharmacopeia. As a consequence, the chemical composition of most NBCDs is only partially discovered. The insufficient chemical identification of the API also makes it difficult to identify other APIs as generics or imitators. In addition, quality and process control may suffer from the absence of a clear chemical description [3, 4].

Bituminosulfonates are APIs derived from kerogen-containing rocks (e.g., organic matter-rich carbonates such as limestone and dolomite^1^). These rocks are mined and subjected to a dry distillation process in order to obtain a sulfur-rich shale oil. Sodium bituminosulfonate (SBS) as investigated herein is obtained by refining, sulfonating, and finally neutralizing the more volatile fraction of the original shale oil with sodium hydroxide. A medium boiling fraction is processed to result in ammonium bituminosulfonate (ABS). Due to origin and course of synthesis, medicinal products including the highly complex substances ABS or SBS can tentatively be allocated to the group of NBCDs today. Its use and efficacy as a therapeutic agent were documented since 1882. Nowadays, research focuses on the pharmacological mechanistic properties of bituminosulfonates at the cellular level [5]. The antimicrobial effects of SBS with low potential to resistance development in bacterial cells are of particular interest [6, 7].

SBS provides several challenges for physicochemical characterization and while the pharmaceutical effects are well documented, the knowledge of the molecular composition is limited. Previous studies were able to identify a small number of adequately chromatographically separable ingredients in the middle distillate fraction. However, this is insufficient to describe the matrix appropriately [8–10]. An additional study from the 1980s investigated the composition of the sulfonated compounds in ABS. In this analysis, sulfonated alkylbenzenes, -thiophenes, and other sulfonated arenes have been detected with a high extent of sample preparation [11]. Recent studies with state-of-the-art instrumentation targeting the chemical composition of the sulfur-rich distillates or their sulfonated products are missing, which limits further development of the API.

Aromatic sulfonates are not very common in industrial processes; just linear alkylbenzene sulfonates are used as detergents [12]. Suitable approved analytical methods and commercially available standard materials are missing. Some targeted analytical methods are reported in literature, e.g., Fourier transform ion cyclotron resonance mass spectrometry (FT-ICR MS) [13], liquid chromatography methods [14], and gas chromatography (GC) in combination with mass spectrometry (MS) [15–17]. Due to their chemical nature, production, and application, the sulfonates are often present in aqueous phase. Therefore, various sample preparation methods and chemical modifications are necessary to make them accessible to some analytical techniques. In particular, a common strategy in GC is to derivatize sulfonates, e.g., with diazomethane [15] or via online derivatization strategies [16, 17]. However, none of these methods provides enough resolution for the analysis of sulfonates derived from shale oil distillates.

Comprehensive two-dimensional gas chromatography (GC × GC) became a broadly used method for the vaporizable fraction of petroleum-derived matrices and was extensively applied for the chemical characterization of shale oils and sulfur-rich petroleum oils [18–21]. GC × GC provides higher chromatographic resolution compared to one-dimensional GC. The combination of various stationary phases allows focusing on specific compound classes in complex mixtures as, e.g., in a study on Estonian shale oils [20] where they analyzed various oxygen-containing compounds with GC × GC and were able to separate them into specific compound classes. Furthermore, sulfur compounds in petroleum matrices are targeted and used as characteristic markers for the geographic and geological origin [19].

Additional selectivity is gained by coupling GC × GC to a selective detector with spectroscopic or spectrometric information. However, the narrow peak width (approx. 50–250 ms), a desired minimum of 10 scans per peak for quantitative description of the peak shape, and potential deconvolution of superimposed peaks limit the selection of detectors and only some types of mass spectrometer (e.g., time-of-flight) or absorbance detectors (e.g., VUV absorption spectroscopy) are fully applicable [22, 23]. Especially mass spectrometers with an electron ionization (EI) source are commonly applied detectors for structural elucidation. EI creates a fragmentation pattern that can be compared to multiple databases and characteristic fragments indicate the presence of functional groups or core structures in the compound. The application of a high-resolution and accurate mass spectrometer (HR-MS) drastically increases the analytical value and adds an additional dimension of information. HR-MS with mass accuracy at ppm levels enables the determination of the elemental composition of the detected ions. The required resolution for separating isobaric compounds depends on the expected elemental compositional space as well as the targeted mass range. Beneficially, the elemental composition of mass fragments related to the intact molecular ion [M]^+●^ can be used to confirm the determined elemental composition [24]. The discrimination of sulfur compounds as dominant ingredients of SBS is a key analytical challenge. In this context, the separation of the 3.4 mDa mass split (^32^SH_4_ versus ^12^C_3_) is of utmost importance but would exceed the mass spectrometric resolving capabilities of most commercially available ToF platforms. However, the combined separation power from chromatography and high mass accuracy and resolution enables the discrimination of sulfur compounds also with limited mass resolution. This facilitates the characterization of samples with high amounts of sulfur with commercially available state-of-the-art GC × GC-HR-ToF–MS [25].

In this study, we describe the application of GC × GC-HR-ToF–MS for the chemical description of an API for complex drugs. Therefore, we analyze the API starting material to identify precursors and present an online derivatization that enables a comprehensive and in-depth chemical characterization of SBS. We show and discuss the as yet unraveled chemical composition of the API in the instrumental context to the applied technique. Moreover, we not only demonstrate the advantages such as the classification of sulfonates by utilizing the accurate mass but also point out the limitations of the application of GC × GC for this API.

## Material and methods

### Sample material and chemicals

Sodium bituminosulfonate (Ichthyol-Natrium Hell®) as well as the unsulfonated API starting materials “vacuum distillate (shale oil), light” (EC 924–360-1; in the following abbreviated as “distillate”) as well as “vacuum distillate (shale oil), light, base and acid treated” (EC 923–317-4; in the following abbreviated as “refined precursor”) from the same production process were provided by Ichthyol-Gesellschaft Cordes, Hermanni & Co. (GmbH & Co.) KG, Germany.

For the online derivatization, tetramethylammonium hydroxide (TMAH) (25% solution in water, Sigma-Aldrich, USA), methanol (> 99.9%, Roth, Karlsruhe, Germany), and purified water (Milli-Q® water purification system (Merck, Darmstadt, Germany)) were used.

### Comprehensive two-dimensional gas chromatography set-up and sample measurement

The GC × GC measurements were carried out on a Pegasus® GC-HRT 4D (LECO, St. Joeseph, USA) with a 60 m BP1 (0.25 mm internal diameter, 0.25 μm film thickness) column in the 1st dimension and 1.5 m BPX50 (0.1 mm internal diameter, 0.1 μm film thickness) in the 2nd dimension. Detailed parameters are listed in Table 1.

**Table 1:** Applied GC × GC parameters for the distillate, refined precursor, and sodium bituminosulfonate (SBS) analysis

	Distillate/refined precursor	SBS
Injection temp [°C]	300	350
Constant flow, helium [ml/min]	1	1
Split	1:200	1:10
Oven program:		
Start temperature	35 °C (5 min)	40 °C (0 min)
Heat rate 1	1 °C/min	10 °C/min up to 120 °C (0 min)
Heat rate 2		1 °C/min
End temperature	235 °C (5 min)	270 °C (0 min)
2nd Oven offset [°C]	55	30
Modulator offset [°C]	15	15
2nd dimension time [s]	5	4.5
Total run time [min]	155	158
Solvent delay [s]	300	1600

One µL of the distillate or the refined precursor were directly injected by hot split injection. For the analysis of SBS, 40 μL were mixed with 80 μL of tetramethylammonia hydroxide, diluted 1:10 with water/methanol (1:1, V/V) and 0.5 μL of the mixture were injected. Five replicates of the distillate and refined precursor were analyzed in randomized order. For the SBS, three replicates were measured.

After every measurement of the SBS, a bake out program with an injection of 0.5 μL derivatization agent was executed, to prevent carry over (330 °C for 15 min). Test blank measurements of the derivatization agent after the measurement sequences were performed to proof absence of carry over (supplementary information (SI): Fig. S1).

Ionization of the eluted compounds was performed by electron ionization with 70 eV at 300 °C. The obtained ions were acquired between *m/z* 15 and 500 in “high-resolution-mode” with an acquisition rate of 100 Hz. Perfluorotributylamine (PFTBA) was continuously added to the MS source as internal standard for mass calibration. Calibration and processing of acquired mass spectra was performed with the ChromaTOF HRT software (v5.10, LECO, St. Joe, USA). During data processing, the experimental masses were calibrated versus the exact theoretical mass information of five known mass fragments of the PFTBA calibrant. A mass resolution of at least 25 k for *m/z* 218.9856 was achieved in all measurements with a mass error below 1 ppm for the standard (SI: Table S1). Peak-picking was applied with a minimum signal-to-noise ratio (S/N) of 10, without a threshold for minimal abundance and with an expected peak width of 0.2 s (SI: Table S2). For further data visualization, a home-built Matlab (R2020b, The MathWorks Inc., MA, USA) script was used.

### Classification

The detected peaks were classified according to their elemental composition (C_c_H_h_N_n_O_o_S_s_). The resulted list of element compositions was further subdivided according to their calculated hydrogen deficiency, displayed as double bond equivalent (DBE):

$$DBE=\frac{2c-h+n+2}{2}$$

The heteroatoms sulfur and oxygen do not contribute to the DBE value. Therefore, the DBE value indicates the hydrogen deficiency at the organic mainly hydrocarbon core structure. Non-hydrocarbons functional groups like sulfonates do not affect the value. Structural classification was performed based on elution behavior and characteristic fragmentation pattern. The classified peaks are further handled as compounds. Although the calibration achieved a mass error below 1 ppm for the classification, a mass tolerance of 5 ppm between the experimental accurate mass and the exact theoretical mass was allowed. This mass window permits a minor difference in the mass spectra by maintaining the distinction of the sulfur split at the observed *m/z* values. NIST (NIST MS Search 2.3, 2017) was applied as reference database for the manual check of plausibility for the classification. The relative abundance of these classes was calculated by the ratio of summed ion count to the overall ion count.

## Results and discussion

### Application of GC × GC-HR-ToF–MS for the chemical description of the process intermediates distillate, refined precursor, and the refining process

The distillate undergoes a refining process, during which it is treated with diluted acid, diluted base, and a solution of minerals. Acidic as well as basic compounds might undergo protonation and deprotonation, respectively, and subsequent elimination from the matrix due to an enhanced solubility in the aqueous phase. In addition, rearrangement and other reactions might occur. The comprehensive chemical description of the distillate and refined precursor allows the investigation of the alteration in the matrix due to this refining process. Knowledge about the chemical composition of the intermediates provides crucial information on the educts for the subsequent sulfonation process to establish a clear educt-product relationship, which could be used for the interpretation of the SBS in the next section.

Like many fossil feedstocks, shale oils exhibit a compositional continuum [26] of organic hydrocarbons and heteroatomic compounds and the distillation discontinues this continuum. The presented boiling range of the distillate (< 300 °C),^2^ as well as the previous thermal treatment by the dry distillation process at 450 °C, leads to the conclusion that the matrix is completely vaporizable without thermal degradation. Therefore, the matrix is suitable and completely covered by GC analysis. Nevertheless, the chromatographic separation has to be sufficient to cope with the complex continuum to allow a subsequent mass spectrometric investigation of individual or resolved chromatographic peaks [25].

In contrast to fossil feedstocks described in the literature [27], a high sulfur content of the unsulfonated API starting materials is has to be considered. Additionally, the presence of unsaturated compounds in these precursor materials is different due to the thermal decomposition of the solid biomass in the preceding manufacturing step [28]. Both aspects present a challenge for the identification via MS. On the one hand, the C_3_–SH_4_ mass split is not resolved by most quadrupole or ToF mass spectrometers [29]. On the other hand, the thermal decomposition process provokes the generation of constitutional isomers such as alkenes/cycloalkanes, which are neither well differentiated by MS nor chromatography [30].

**Fig. 1:**
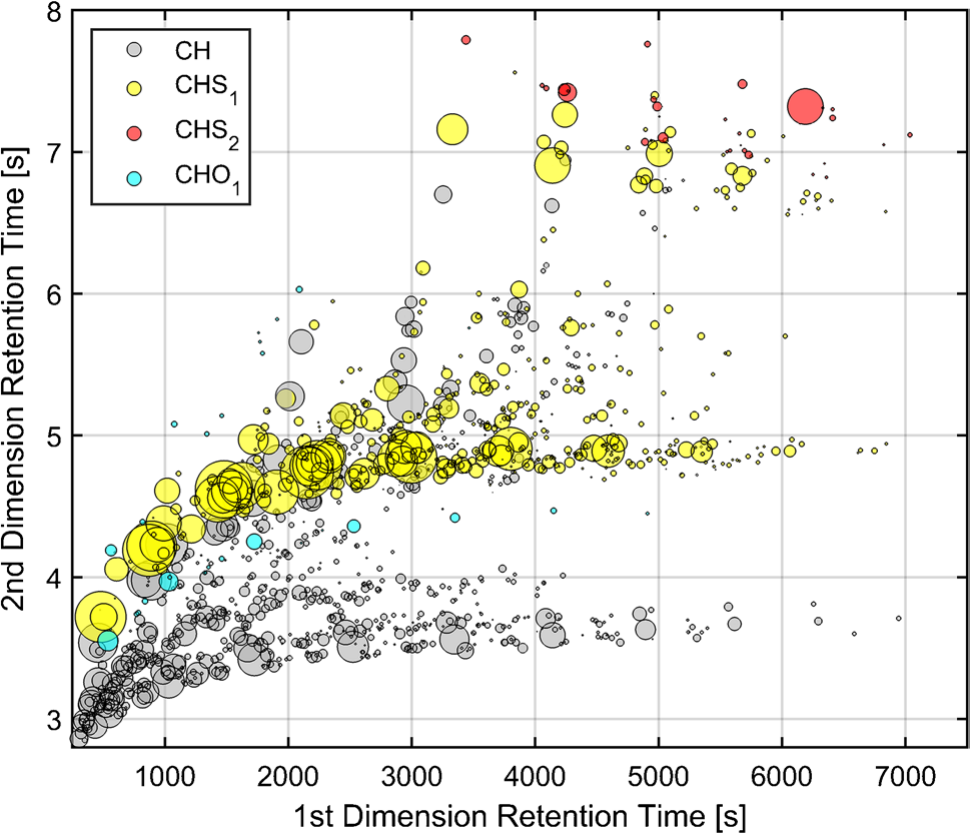
GC × GC-HR-ToF–MS scatter plot for the classified peaks found in the refined precursor (visual shift of the second dimension: 2.8 s). The color code of the bubbles refers to their elemental composition and their volume to the abundance of their summed ion count (contour plot SI: Figs. S2 and S3)

The observed chromatographic pattern, depicted from the two-dimensional visualization of the classified peak distribution, described a characteristic elution profile similar to fossil continuums (Fig. 1). The chromatographic separation was performed on a non-polar column in the first dimension, which resulted in a simulated distillation and respective elution order of the compounds [31]. Therefore, the homologous rows were separated according to their carbon number. The mid-polar stationary phase in the second dimension exhibited π-π interactions as main retention mechanism in the quasi-isotherm temperature profile of the modulation step. Hence, the detected compounds increased in polarizability and aromaticity with higher retention times. On average, 1903 peaks in the refined precursor and 2061 in the distillate were detected with a S/N value above 100 (SI: Tables S3 and S4).

**Fig. 2:**
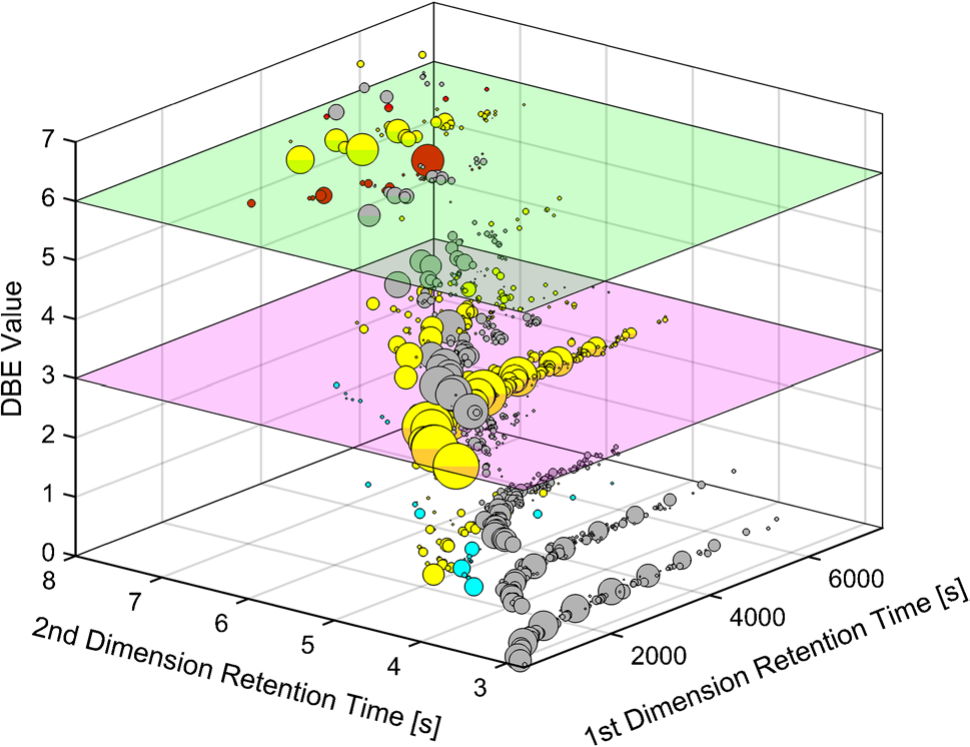
GC × GC-HR-ToF–MS 3D scatter plot of the refined precursor for the classified peaks with further discrimination of the chemical classification by integration of the hydrogen deficiency (DBE) (Fig. 1) (visual shift of the second dimension: 2.8 s). Prominent DBE classes (DBE 3 (purple) and DBE 6 (green)) which are further discussed are highlighted. The color code of the bubbles refers to their elemental composition (CH: grey, CHO_1_: blue, CHS_1_: yellow, and CHS_2_: red) and their volume to the abundance of their summed ion count (contour plot SI: Figs. S2 and S3)

Peaks were first assigned to their elemental classes into pure hydrocarbons (C_c_H_h_), nitrogenous organic compounds (CHN_1_ = C_c_H_h_N_1_), oxygenated organic compounds (CHO_1_ = C_c_H_h_O_1_), and sulfurous organic compounds (CHS_1_ and CHS_2_ = C_c_H_h_S_1_ and C_c_H_h_S_2_) (Fig. 1). Other elemental classes with a combination of heteroatoms or higher numbers were also considered but they were not detected or only in a small extent (< 5 peaks and < 0.1% rel. abundance). A further subdivision according to hydrogen deficiency with the DBE as integer value (Fig. 2) is listed in Table 2. This mass spectrometric discrimination of the classes is based on accurate mass information of the molecular ions [M]^+●^ and fragments. Further structural classification of the found elemental compositions was based on the fragmentation pattern and the elution profile. The fragmentation pattern of aromatic compounds often provides information about the aromatic core structure, which is energetically favored (stabilized) during fragmentation and carries the positive charge. The heterocyclic core is also often relatively stable and a carrier of the positive charge. These energetically stabilized structures are very dominant (e.g., base fragment) in the spectra (Table 2) and can be used for classification. If sufficient chromatographic separation was already achieved, additional non-characteristic but abundant fragments could be used for the assignment of the peaks. The presence of overlapping elemental classes with different elemental compositions required the use of the accurate mass information of the fragments to achieve sufficient discrimination with a maximum mass tolerance of ± 5 ppm.

**Table 2:** List of substance classes found in the API and its process intermediates. Classes are written in italic, if multiple structural classes were possible and/or a clear assignment was not possible. Exact masses of the molecular ions [M]^+●^ (if applicable, first row of the cell) and of the characteristic fragments, rel. abundances, and relative standard deviation are given (raw data in SI: Table S5, Table S6, and Table S7). *N.D.*, not detected

No	Structural classes	Elemental classes		Exact masses [Da] used for classification ± 5 ppm	Average rel. abundance [%] (relative standard deviation [%])		
		Elements	DBE		Distillate	Refined precursor	SBS^1^
I	Alkanes	CH	0	43.0542, 57.0699, 71.0855, 85.1012	4.3 (± 3.8)	4.7 (± 1.6)	N.D
II	*Cycloalkanes, Alkenes*	CH	1	41.0386, 55.0542, 69.0699, 83.0855	8.0 (± 8.0)	8.8 (± 2.6)	N.D
III	*Dicycloalkanes, Dialkenes, Cycloalkanes, Alkynes*	CH	2	67.0542, 81.0699, 95.0855	10.4 (± 3.7)	10.7 (± 3.2)	N.D
IV	*Multiple combination of double bounds and cycles*	CH	3	79.0542, 93.0699, 107.0855	3.7 (± 6.5)	2.5 (± 5.2)	N.D
V	Benzenes	CH	4	[M]^+●^ 78.0464, 92.0621, 106.0777, 120.0934, 134.1090 91.0542, 105.0699, 119.0855, 133.1012	6.9 (± 1.4)	7.3 (± 2.9)	20.7 (± 2.0)
VI	Indanes	CH	5	[M]^+●^ 118.0777, 132.0934, 146.1090, 160.1247 117.0699, 131.0855, 145.1012, 159.1168	3.3 (± 3.1)	3.9 (± 8.1)	1.9 (± 8.8)
VII	*Indenes, Dialkenylbenzenes, Alkenylindans*	CH	6	[M]^+●^ 116.0621, 130.0777, 144.0934, 158.1090 115.0542, 129.0699, 143.0855, 157.1012	2.9 (± 6.1)	3.2 (± 1.4)	N.D
VIII	Naphthalenes	CH	7	[M]^+●^ 128.0621, 142.0777, 156.0934 141.0699, 155.0855	0.3 (± 9.5)	0.4 (± 8.3)	0.3 (± 0.8)
IX	Thiophanes	CHS_1_	1	[M]^+●^ 88.034, 102.0498 60.002, 87.0263, 101.0419	1.7 (± 9.0)	1.8 (± 6.5)	N.D
X	Didehydrothiophanes	CHS_1_	2	[M]^+●^ 86.0185, 100.0341 85.0106, 99.0263	0.4 (± 6.1)	0.1 (± 39.3)	N.D
XI	Thiophenes	CHS_1_	3	[M]^+●^ 84.0028, 98.0185, 112.0341, 126.0498, 140.0654 97.0106, 111.0263, 125.0419, 139.0576	25.2 (± 2.0)	26.4 (± 2.0)	65.0 (± 3.0)
XII	*Tetrahydrobenzo-thiophenes, Benzenethiols/-thioethers, Alkenylthiophenes*	CHS_1_	4	[M]^+●^ 110.0185, 124.0341, 138.0498, 152.065, 166.081 123. 0263, 137.0419, 151.058, 165.073	6.3 (± 1.9)	6.1 (± 4.7)	2.8 (± 8.9)
XIII	*Dihydrobenzothiophenes, Phenylthiophanes*	CHS_1_	5	[M]^+●^ 150.0498, 164.654, 178.0811 149.0419, 163.0576, 177.0732	0.1 (± 12.0)	0.4 (± 5.0)	N.D
XIV	Benzothiophenes	CHS_1_	6	[M]^+●^ 134.0185, 148.0341, 162.0498, 176.065 147.0263, 161.0419, 175.0576	2.6 (± 3.9)	2.8 (± 1.6)	1.6 (± 0.9)
XV	Phenylthiophenes	CHS_1_	7	[M]^+●^ 160.0341, 174.0498 173.0419	0.1 (± 22.6)	0.1 (± 22.1)	< 0.1 (± 4.9)
XVI	Thienothiophenes	CHS_2_	5	[M]^+●^ 139.9749, 153.9905, 168.0062 152.9827, 166.9984	1.9 (± 63.3)	0.7 (± 41.9)	N.D
XVII	Bithiophenes	CHS_2_	6	[M]^+●^ 165.9905, 180.0062, 194.0218 178.9984, 193.0140	0.9 (± 122.4)	0.2 (± 6.4)	0.1 (± 30.2)
XVIII	Aliphatic Ketones	CHO_1_	1	[M]^+●^ 58.0413 43.0178	0.8 (± 2.2)	0.8 (± 5.3)	N.D
XIX	*Cycloalkanones, Alkenones*	CHO_1_	2	55.0178	0.1 (± 7.6)	0.1 (± 2.6)	N.D
XX	Cyclopentenones	CHO_1_	3	[M]^+●^ 82.0413 67.0542	0.1 (± 3.1)	< 0.1 (± 12.3)	N.D
XXI	Phenols	CHO_1_	4	[M]^+●^ 94.0413, 108.0570, 122.0726 107.0491, 121.0648	0.3 (± 11.7)	< 0.1 (± 68.7)	N.D
XXII	Pyrroles	CHN_1_	3	[M]^+●^ 67.0417, 81.0573, 95.073 80.0495, 94.0651	< 0.1 (± 24.8)	N.D	N.D
XXIII	Pyridines	CHN_1_	4	[M]^+●^ 79.0417, 93.0573 92.0495	0.1 (± 5.2)	N.D	N.D

^1^Sulfonated product (elemental classes + O_3_S).

In most cases, the chromatographic and mass spectrometric information enabled not only the discrimination of elemental classes but also the assignment of structural classes. In the following, the identified structural classes are depicted in brackets. Structural classes, which could not be assigned clearly, are depicted in Table 2 in italics. The classes mentioned in the main text refer to Table 2 by the number of the row in roman numbers. In total, the classification covered the majority of all peaks (81.3% of the overall number of peaks (SI: Table S3) and 81.1% of the summed ion count (SI: Table S5) in the refined precursor).

In general, sulfur-containing aromatic classes (CHS_1_ DBE 3–7, XI–XV) were very prominent and CHS_1_ DBE 3 (thiophenes, IX) could be identified as the most abundant class (Table 2). Other abundant classes were CH DBE 4 (benzenes, V), CH DBE 5 (indanes, VI), CH DBE 6 (VII), CH DBE 7 (naphthalenes, VIII), CHS_2_ with DBE 5 (thienothiophenes, XVI), and DBE 6 (bithiophenes, XVII). Especially these aromatic compounds provide a high reactivity towards a subsequent sulfonation reaction. The applied technique allowed the assignment of elemental composition, hydrogen deficiency, and structural information for most of these classes.

However, it was not possible to attribute the exact structural class for all found elemental compositions by GC × GC-HR-ToF–MS. The elemental class CHS_1_ DBE 4 (XII) is one example for the difficulties of an exact structural classification. For the attributed elemental class, different isomeric compositions of the core-structural motif are possible. Unfortunately, the elution behaviors, as well as fragmentation pattern, of these structures are very similar. In addition, only a limited number of smaller derivatives are found in MS libraries. Therefore, the elemental class could not be uniquely attributed to one structural class and is tentatively described as group of tetrahydrobenzothiophenes, benzenethioles/-thioethers, and alkenylthiophenes (XII).

In general, the relative standard deviation (RSD) for the measurement of the replicates was below 10% (with relative abundance > 0.5%). Only the two CHS_2_ classes (XVI and XVII) showed a RSD > 50% in combination with a relative abundance above 0.5%. The peaks of both classes were partly co-eluting with column bleed, which was not completely deconvoluted and, thus, partly included in the summed mass spectra of the peaks. The use of characteristic fragments for obtaining quantitative information takes this into account. For the lower abundant compound classes like phenols (XXI) also higher RSD values were calculated. This could be explained by the higher influence of small altering effects from the analysis to the low abundant peaks near the limit of detection. Due to the low RSDs for most of the classes, a high precision in reference to repeatability was achieved. Therefore, the requirements for a comparison of the distillate and the refined precursor were met.

The overall pattern (SI: Figs. S2, S3, S4, and S5), as well as the integrated values (Table 2), for the different classes found in refined precursor and distillate was qualitatively and quantitatively similar. Significant differences between the refined precursor and distillate appeared only for less abundant classes like CHO_1_ DBE 4 (phenols, XXI), CHN_1_ DBE 4 (pyridines, XXIII), and CHN_1_ DBE 3 (pyrroles, XXII). All these classes were depleted in the refined precursor, the latter below the limit of detection. Compounds of these classes are most likely sensitive towards the treatment with acids respectively bases and can subsequently be removed with the aqueous phase. Compounds like phenol or pyridine that are contained in these classes affect the human health and their elimination in the refining process is beneficial. The refining process also showed an effect in the CH DBE 3 (III), CHS_1_ DBE 2 (X), and CHS_1_ DBE 5 (XIII) groups. However, it was not yet possible to evaluate a reason for these behaviors.

In summary, the application of GC × GC-HR-ToF–MS allowed us to cover and describe most of the matrix. About 80% in terms of peak number and summed peak abundance found in the distillate and refined precursor could be assigend. Compared to the most comprehensive previous studies of Pailer et al. [8–10] that needed multiple separation steps and identified about 100 compounds, the GC × GC method did not need any sample treatment and was able to classify more than 1500 peaks. The vast majority of the matrix could be assigned to the chemical classes of CH DBE 0–7 (I–XIII) and CHS_1_ DBE 1–7 (IX–XV). Most of the classes could be assigned to their core-structural motif, while others could be at least linked to an elemental composition with the DBE as hydrogen deficiency measure. It was also possible to reveal the chemical changes induced by the refining process, in particular, the reducing effect to polar substance classes like CHO_1_ DBE 4 (phenols, XXI) and CHN_1_ DBE 4 (pyridines, XXIII). Therefore, the refining process eliminated these potential harmful compounds, while the overall chemical composition of pure hydrocarbons- and sulfur-containing arenes was not altered.

### Application of an online derivatization GC × GC-HR-ToF–MS method for the detailed chemical description of the active pharmaceutical ingredient sodium bituminosulfonate

The batch sulfonation of the refined precursor results in SBS. The strongly exothermic reaction of the electrophilic aromatic substitution requires temperature control and cooling. The raw product is neutralized with a sodium hydroxide solution and decanted, to separate the water soluble and non-soluble phase. The reactivity of the mainly aromatic educts differs depending on the chemical functionality, elemental composition, aromaticity, and the position and properties of substituents. The main reaction pathway for the most abundant constituents results in aromatic sulfonic acids, which are present in the aqueous phase [32]. The predominantly lipophilic and apolar components of the starting materials remain in the non-aqueous organic phase. The formed sulfonic acids are strong acids (e.g., pKa of − 2.8 for benzenesulfonic acid), hygroscopic, and known as detergents [33].

**Fig. 3:**
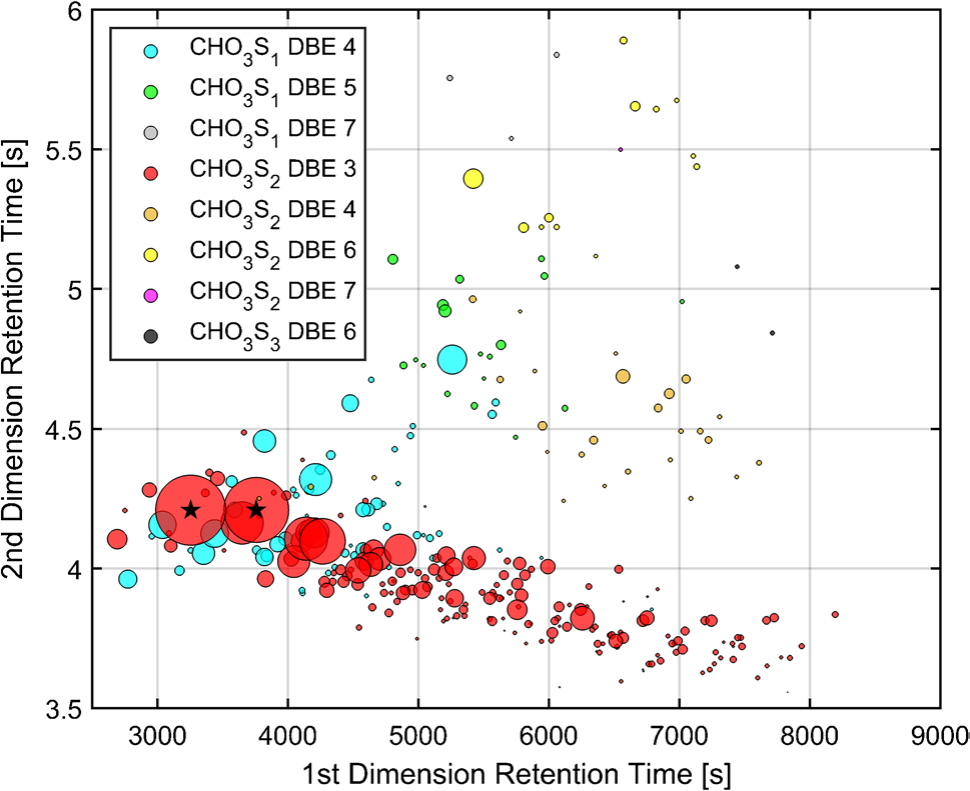
GC × GC-HR-ToF–MS scatter plot for the classified peaks found in the SBS (visual shift of the second dimension: 1.8 s) (1st dimension: 2700–9000 s; 2nd dimension: 3.5–6 s; complete contour plot SI: Figs. S6 and S7). The two most abundant peaks are labeled with a black star. The color code of the bubbles refers to their elemental class (Table 2) and their volume to their abundance

Unlike refined precursor and distillate, the sulfonated salts are not decomposition-free evaporable and are therefore not directly accessible by GC. An additional challenge for further sample preparation is the poor solubility of the SBS in common organic solvents, which complicates the removal of water. For example, dried SBS could only be dissolved in aqueous mixtures (e.g., methanol:water, 1:1 vol%). To convert the salt into a vaporizable GC-accessible product, an online derivatization with TMAH in methanol:water was established. Consequently, the sulfonates were converted into temperature-stable and vaporizable methyl esters. At first glance, the elution behavior of the derivatized products already indicated more polar and heavier compounds compared to the educts found in the refined precursor (Fig. 3).

Most reactive precursor compounds, as well as aliphatic compounds, were depleted or below the detection limit. The obtained mass spectra indicate predominantly sulfonated compounds, which could be evaluated due to accurate mass information of the found molecular ions and by comparing the fragmentation pattern of benzene-, toluene-, and naphthalene sulfonic acid methyl ester to the NIST database.

Nevertheless, the classification of peaks from sulfonated compounds presented greater challenges than for their non-sulfonated precursors, because of the sulfonic group that influences the fragmentation behavior, causing rearrangements that are dependent on the substitution pattern [34]. Therefore, the classification for sulfonated elemental classes used the molecular ion [M]^+●^, which was present for all aromatic classes with a rel. abundance > 10% in the mass spectra of the peak. Taking this as a starting point, the continuous increase of the masses by an equivalent of CH_2_ for the alkylated homologue series was used subsequently for the assignment (SI: Fig. S6).

Similar to the API starting materials, the combination of the accurate mass and elution pattern enabled the classification of the vast majority of peaks into elemental and structural classes. The classified peaks represented about 70% (SI: Table S8) of the overall detected number of peaks and more than 85% of the overall peak abundance (SI: Table S9). The main part was assigned to mono-sulfonated arenes (SI: Table S7). Eight sulfonic elemental classes with a total of more than 500 peaks were classified. The formula for the calculation of the DBE value does not include the double bonds in the sulfonate group (R-SO_3_-R). Therefore, the DBE value of the sulfonated elemental classes was a measure of hydrogen deficiency for the core structure. This allowed a direct comparison to the precursor constituents found in the refined precursor. Thus, seven elemental classes were also assigned to structural classes; just the elemental class of CHO_3_S_2_ DBE 4 (XII) could not be uniquely identified.

**Fig. 4:**
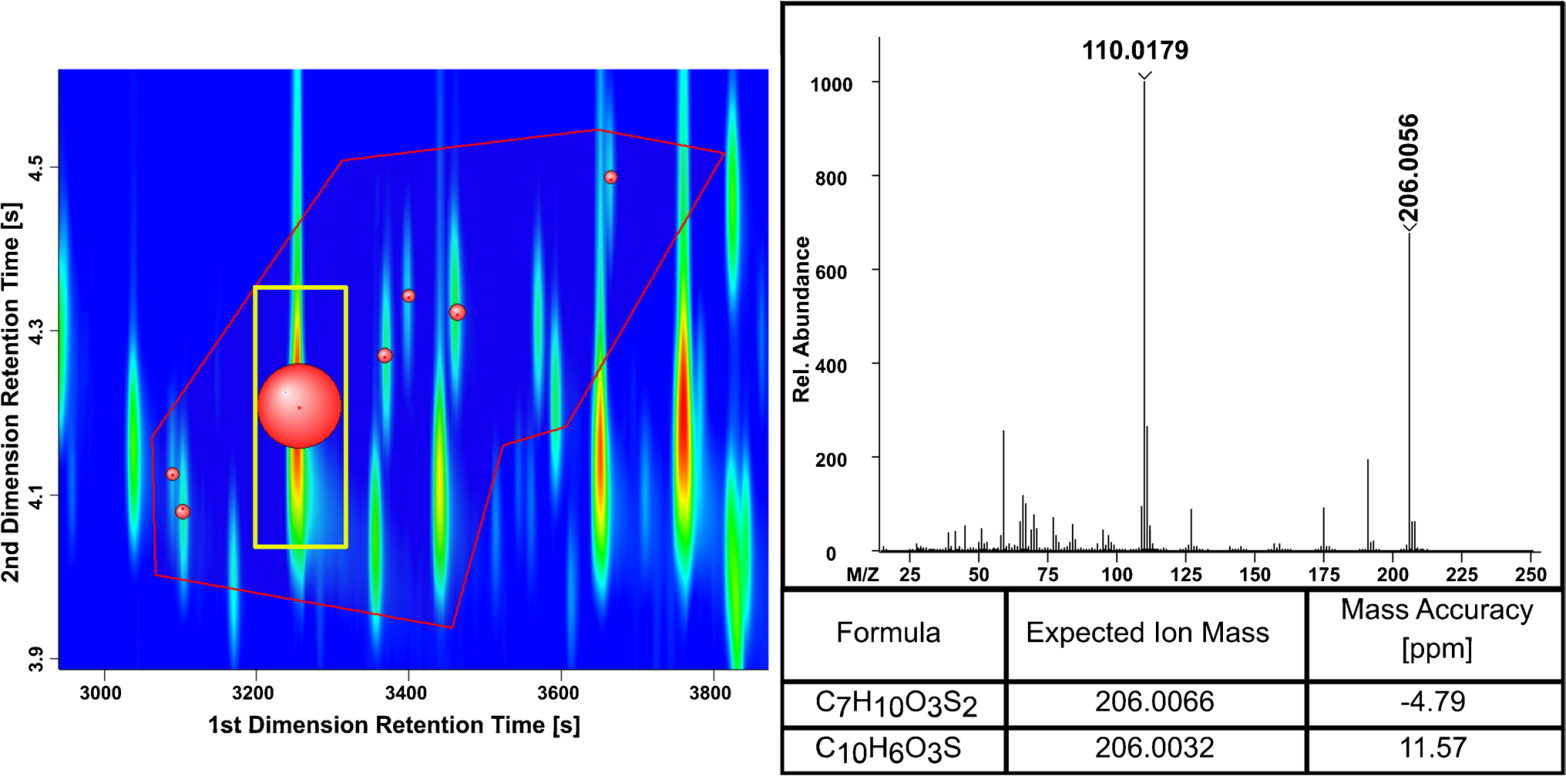
Section of an GC × GC-HR-ToF–MS contour plot of SBS (visual shift of the second dimension: 1.8 s) (1st dimension: 2900–3900 s; 2nd dimension: 3.85–4.6 s; complete contour plot SI: Figs. S6 and S7) and mass spectra of the most abundant peak with the assigend sum formula for a mass accuracy < 5 ppm and the most plausible alternative (mass accuracy) with the elemental composition of ^12^C ≤ 50, ^1^H ≤ 100, ^16^O ≤ 7, ^32^S ≤ 6, and ^14^ N = 0. Labeled peaks inside the elution region (red frame) provide the similar molecular ion [M]^+●^. The volume of the peak bubbles is dependent on their relative abundance

The derived accurate masses for the SBS presented a greater mass error than the standards in the calibration indicated. However, even at differences close to 5 ppm, the elemental composition for sulfonates could be assigend (Fig. 4). The presence of additional compounds with the same mass for their molecular ion [M]^+●^ (within the mass tolerance) and with a similar elution confirmed the assignment of the elemental composition. Moreover, the assignment was confirmed by the isotop ratio of ^32^S and ^34^S ([M]^+●^ and [M + 2]^+●^) (SI: Fig. S8). Another level of confidence was given by the analysis of the refined precursor as educt for the API. Furthermore, also the knowledge about the production process limited the possible elemental composition to carbon, hydrogen, oxygen, and sulfur.

The most abundant class was CHO_3_S_2_ DBE 3 (sulfonated thiophenes, XI), which presented more than half of the classified peaks in terms of number and area percentage (Table 2 and Fig. 3). Other dominant substance classes were CHO_3_S_1_ DBE 4 (sulfonated benzenes, V), CHO_3_S_2_ DBE 4 (XII), and CH_3_O3S_2_ DBE 6 (sulfonated benzothiophenes, XIV). Their share decreased in the listed order and other sulfonated compounds like CHO_3_S_1_ DBE 5 or 7 (sulfonated indanes and naphthalenes, VI and VIII) or CHO_3_S_3_ DBE 6 (sulfonated bithiophenes, XVII) showed just a small extent with relative abundance below 1% and 0.1%, respectively.

Almost all aromatic classes present in the refined precursor could be found as sulfonated reaction products in the SBS matrix. However, the sulfonated products of CH DBE 6 (VII) or CHS_2_ DBE 5 (thienothiophenes, XVI) were not detected in the SBS, while the lower abundant structural classes with higher DBE (naphthalenes and bithiophenes, VIII and XVII) were present. One possible explanation is a poorer reactivity of these two classes in combination with their small abundance in the refined precursor. However, due to limited literature regarding the reactivity of these substance classes towards sulfuric acid, this could not be confirmed. The other elemental classes of the sulfonated product can be found in a similar order of their abundance distribution compared to their non-sulfonated precursor classes in the API starting materials (Table 2). Although the sulfonation and the washing step removed non-aromatic constituents, which were not sulfonated, the relative abundance of CHO_3_S_2_ DBE 3 (sulfonated thiophenes, XI) and CHO_3_S_1_ DBE 4 (sulfonated benzenes, V) increased. The high abundance of the CHO_3_S_2_ DBE 3 (sulfonated thiophenes, XI) could be explained by the high reactivity of the CHS_1_ DBE 3 (thiophenes, XI) precursor in the starting material. However, benzene is less reactive than naphthalene and as reactive as benzothiophenes but their alkylation could support the sulfonation and increase the reactivity [32, 33].

Beside the sulfonated elemental classes, just a small extent of sulfur containing non-sulfonated classes including CHS_1_ DBE 3 (thiophenes, XI) were found in SBS. However, the absence of substance classes with poor reactivity such as non-sulfonated benzenes could be also an indication for a thermal degradation due to the high injection temperatures (350 °C). Because of the low extent of these classes and missing literature to evaluate the thermal stability of sulfonated thiophenes, they were not further investigated in this study.

The elemental composition of the molecular ion was further used to separate the elemental classes according to their carbon number (SI: Fig. S6, Tables S8 and S9). Two peaks for the sulfonated thiophenes were dominant, one with seven and the other with eight carbon atoms (Fig. 3, highlighted with a black star). Therefore, taking into account the core-structural motif, they were assigned as compounds with two, respectively three carbons in the alkyl substitution at the thiophene core. A further identification was not possible due to the lack of standards and database hits. Because of their high abundance, they lead to a strong tailing in the second dimension resulting in an underrepresentation of their abundances due to processing issues.

Although SBS presented more challenges than its precursor, the used GC × GC-HR-ToF–MS method with online derivatization enabled the analysis. The results of this method presented the expected sulfonated substance classes of the refined precursor with CHO_3_S_2_ DBE 3 (sulfonated thiophenes, XI) as the most abundant class, followed by CHO_3_S_1_ DBE 4 (sulfonated benzenes, V), CHO_3_S_2_ DBE 4 (XII), and CHO_3_S_2_ DBE 6 (sulfonated benzothiophenes, XIV). Additionally, the most characteristic peaks could be determined as C_2_ and C_3_ alkylated sulfonated thiophenes. Besides the mono-sulfonated substance classes, just a small extent of non-sulfonated substance classes were detected. Koch et al. [11] analyzed a comparable matrix using 1D GC but their results are limited to a list of compound classes and their degree of alkylation without chromatographic information. Therefore, a direct comparison was not possible but the high number of peaks, their similar polarity, and size would be unresolvable by a 1D GC.

## Conclusion

The study presents the benefits of the application of GC × GC-HR-ToF–MS for the chemical description of complex drugs. It was possible not only to describe the process intermediates, which presented boiling points below 300 °C, but also SBS was measureable with its expected sulfonated substance classes.

The presented GC × GC method provided a high repeatability and was able to identify small differences induced by the refining process. Due to the high mass resolution and accuracy in combination with the elution profile, it was possible to classify the detected peaks by elemental composition and to assign them to the underlying structural classes. The use of DBE together with the information on the elemental composition allowed the creation of an additional dimension to discriminate the found classes (Fig. 2). As a result, about 80% of the summed peak area and summed peaks number found in the starting materials distillate and refined precursor could be assigned, with the structural class of the thiophenes as the most abundant.

Although SBS provides greater challenges in terms of sample preparation and classification, the presented online derivatization method achieved a similar repeatability as for the precursor. It was possible to classify more than 85% of the detected matrix and to assign over 500 peaks to eight sulfonated elemental classes. Even without purchasable standards and with a limited database, seven of these classes could be also assigned to structural classes. The stability of the molecular ion [M]^+●^ of the sulfonated compounds enabled a discrimination of the substance classes to their carbon number and confirms the elution region. This method not only identified sulfonated thiophenes and sulfonated benzenes as most intense classes but also revealed two very prominent peaks, one C_2_ and the other C_3_ alkylated sulfonated thiophene.

The high extent of detected and assigned species emphasize the difficulties in the analysis of complex drugs, where commonly used methods reach their limits. However, the powerful analytical technique of online derivatization GC × GC-HR-ToF–MS enabled the in-depth analysis of the API sodium bituminosulfonate. Although SBS was approved decades ago, the shown results are crucial to comply with regulatory requirements and providing more detailed chemical information on the well-established complex API to facilitate future downstream product development. Additionally, the method provides the basis for the differentiation from competitors and copycat products.

Nevertheless, analyses for pharmaceuticals have to work according to high standards to ensure the validity of their results. Therefore, the evaluation of the analysis according to standards of the pharmaceutical industry by comparing the results to other methods would be the next step to narrow the gap between academic research and the industrial implementation.

## Supplementary Information

Below is the link to the electronic supplementary material.

Supplementary file1 (DOCX 32.6 MB)

## Footnotes

1 This research work refers to bituminosulfonates under the trademark Ichthyol®.

2 Presented by a boiling point analysis of the Ichthyol-Gesellschaft.
