# Supplementary material for: Analysis of complex drugs by comprehensive two-dimensional gas chromatography and high-resolution mass spectrometry: detailed chemical description of the active pharmaceutical ingredient sodium bituminosulfonate and its process intermediates
Source: Anal Bioanal Chem. 2022 Nov 19;415(13):2471–81. doi: 10.1007/s00216-022-04393-w (PMC10149445; doi:10.1007/s00216-022-04393-w)
Supplement: Supplementary file 1 — Supplementary file1 (DOCX 32.6 MB) [file 216_2022_4393_MOESM1_ESM.docx]

**Supplementary Information:**

**Analysis of Complex Drugs by Comprehensive Two-Dimensional Gas Chromatography and High-Resolution Mass Spectrometry: Detailed Chemical Description of the Active Pharmaceutical Ingredient Sodium Bituminosulfonate and its Process Intermediates**

Schwalb, L.^1,2^; Tiemann, O.^1^; Käfer, U.^1,2,3^; Gröger, T.^2,*^; Rüger, C. P.^1^; Gayko, G.^4^; Zimmermann, R.^1,2^

^1^ Joint Mass Spectrometry Centre (JMSC), Chair for Analytical Chemistry, University of Rostock, Rostock, Germany

^2^ Joint Mass Spectrometry Centre (JMSC), Cooperation group “Comprehensive Molecular Analytics” (CMA), Helmholtz Zentrum München GmbH, German Research Center for Environmental Health, Neuherberg, Germany

^3^ now at: Leibniz-Institute for Tropospheric Research (TROPOS), Leipzig, Germany

^4^ Ichthyol-Gesellschaft, Cordes, Hermanni & Co. (GmbH & Co.) KG, Hamburg, Germany

*Corresponding Author: Gröger, Thomas <thomas.groeger@helmholtz-muenchen.de>

Keywords: GC×GC, HR-MS, Classification, Non-Biological Complex Drugs, Complex Drugs, Sodium Bituminosulfonate

The Parameters for the distillate and SBS were adapted to the physical properties of the matrix. The column combination, the temperature program, the offset and the second dimension time presented the best-tested chromatographic separation for the majority of the peaks. The injection temperature was investigated as well. The boiling point range of the precursors mainly determined their injection temperature (300 °C). For the SBS a higher temperature was needed to ensure the fast online derivatization. At lower temperatures, the derivatized peaks presented an extensive tailing.

The measurement of the distillate was repeated regularly and checked visually to observe the quality of the chromatographic separation. Here, no major differences in the retention times, sensitivities and chromatographic resolutions were observed.

The depicted chromatograms were visually shifted to avoid wrap around and to facilitate the interpretation of the polarities. Therefore, the shifted time was removed from the bottom of the chromatogram and added on the top.


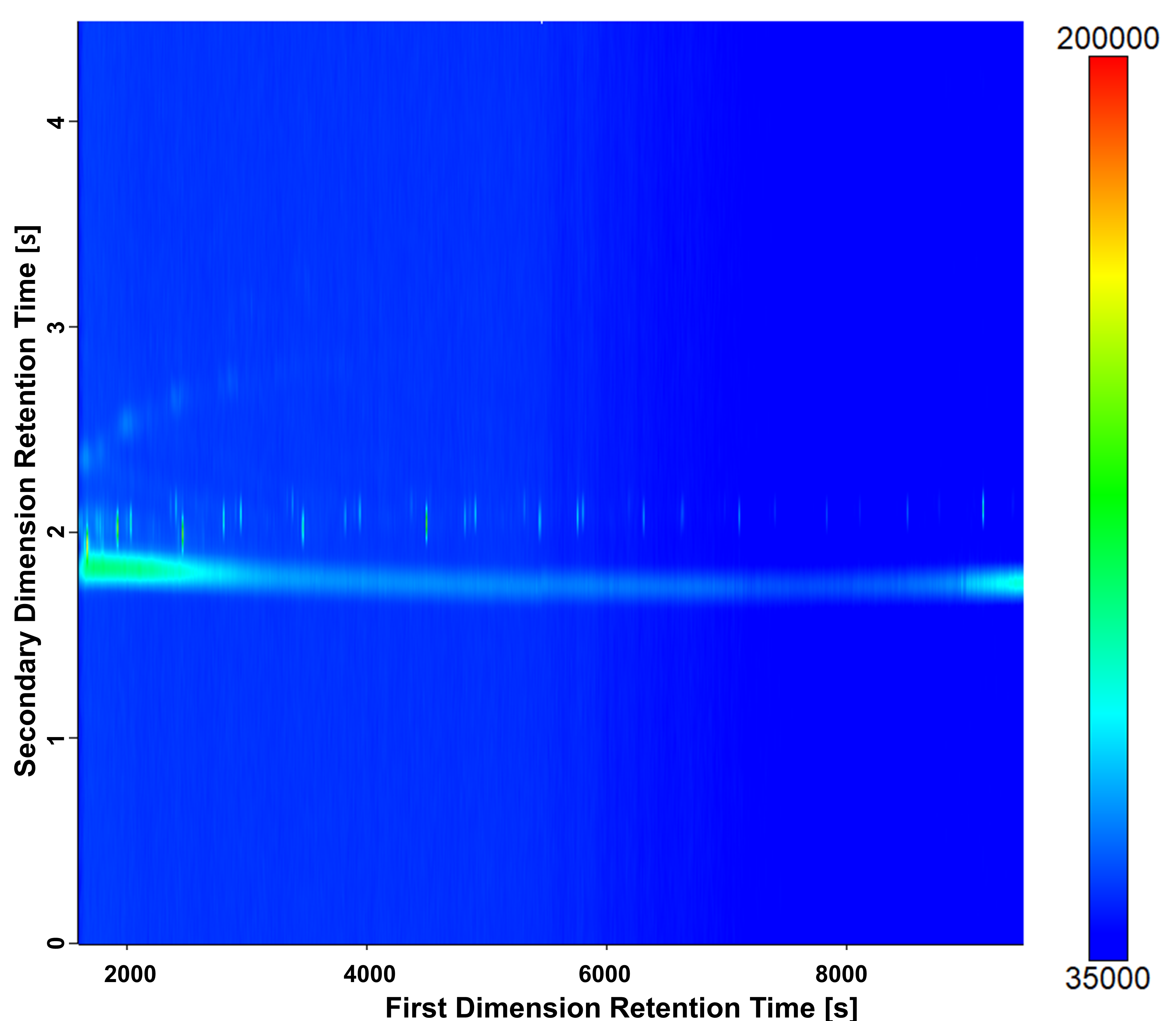


*Fig S1 GC×GC-HR-ToF-MS contour plot for the blank measurement of the derivatization agent after the measurement of SBS.*

*Fig S2 GC×GC-HR-ToF-MS contour plot for refined precursor (Visual shift of the second dimension: 2.8 s). Only classes and their elution range are indicated, which are discussed in the text. The color code of the peaks refers to their elemental composition (raw contour plot Fig S3).*

*Fig S3 GC×GC-HR-ToF-MS contour plot for refined precursor without visual modification.*


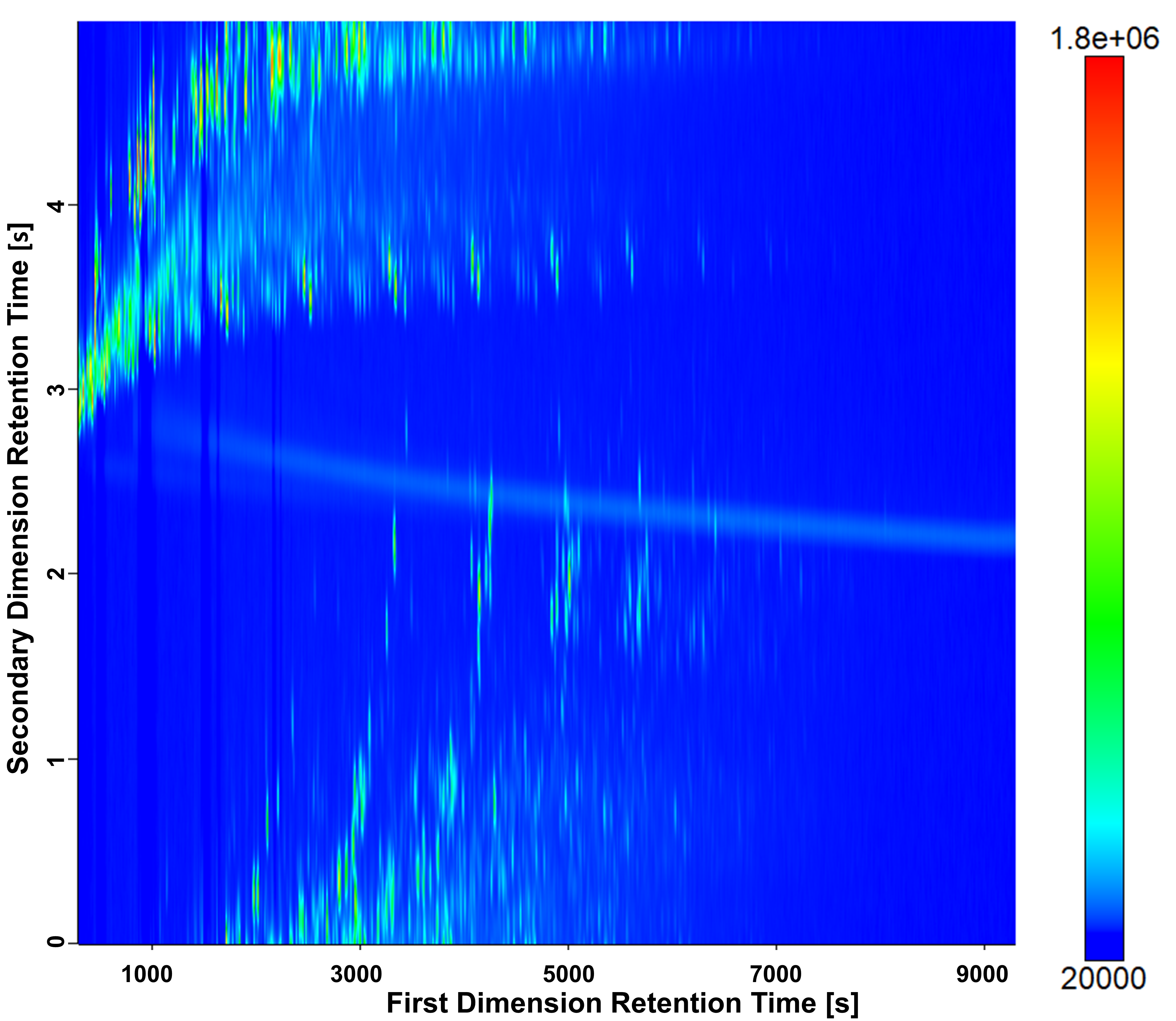

Fig S4 GC×GC-HR-ToF-MS contour plot for distillate (Visual shift of the second dimension: 2.8 s). Only classes and their elution range are indicated, which are discussed in the text. The color code of the peaks refers to their elemental composition.

*Fig S5 GC×GC-HR-ToF-MS contour plot for distillate without visual modification.*


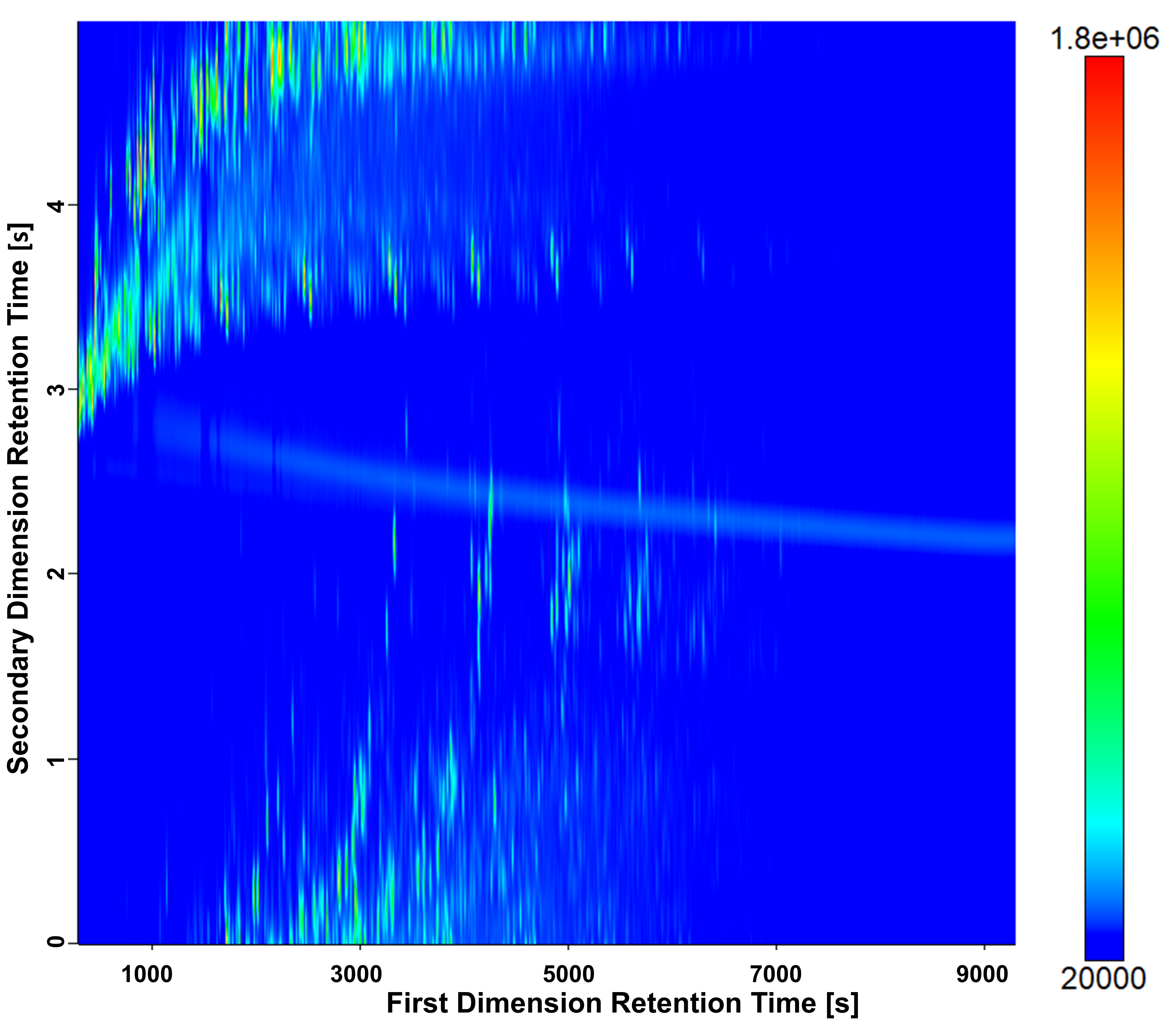

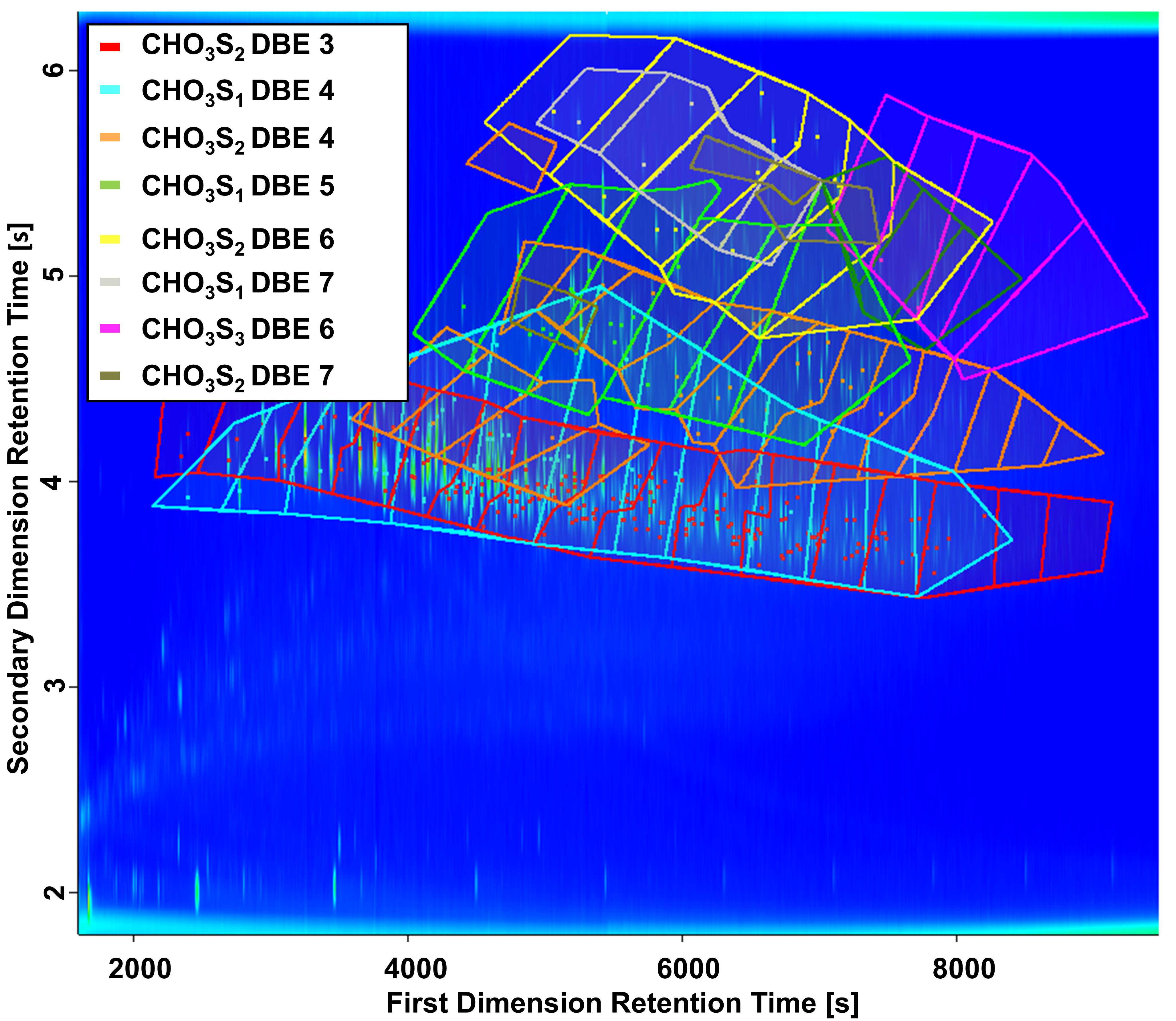


Fig S6 GC×GC-HR-ToF-MS contour plot of SBS (Visual shift of the second dimension: 1.8 s). General classification (color) is based on DBE and elemental composition and was further subdivided into ascending Carbon numbers. The peaks in the column bleed and other artifacts are excluded from the statistically used area.

*Fig S7 GC×GC-HR-ToF-MS contour plot for SBS without visual modification.*


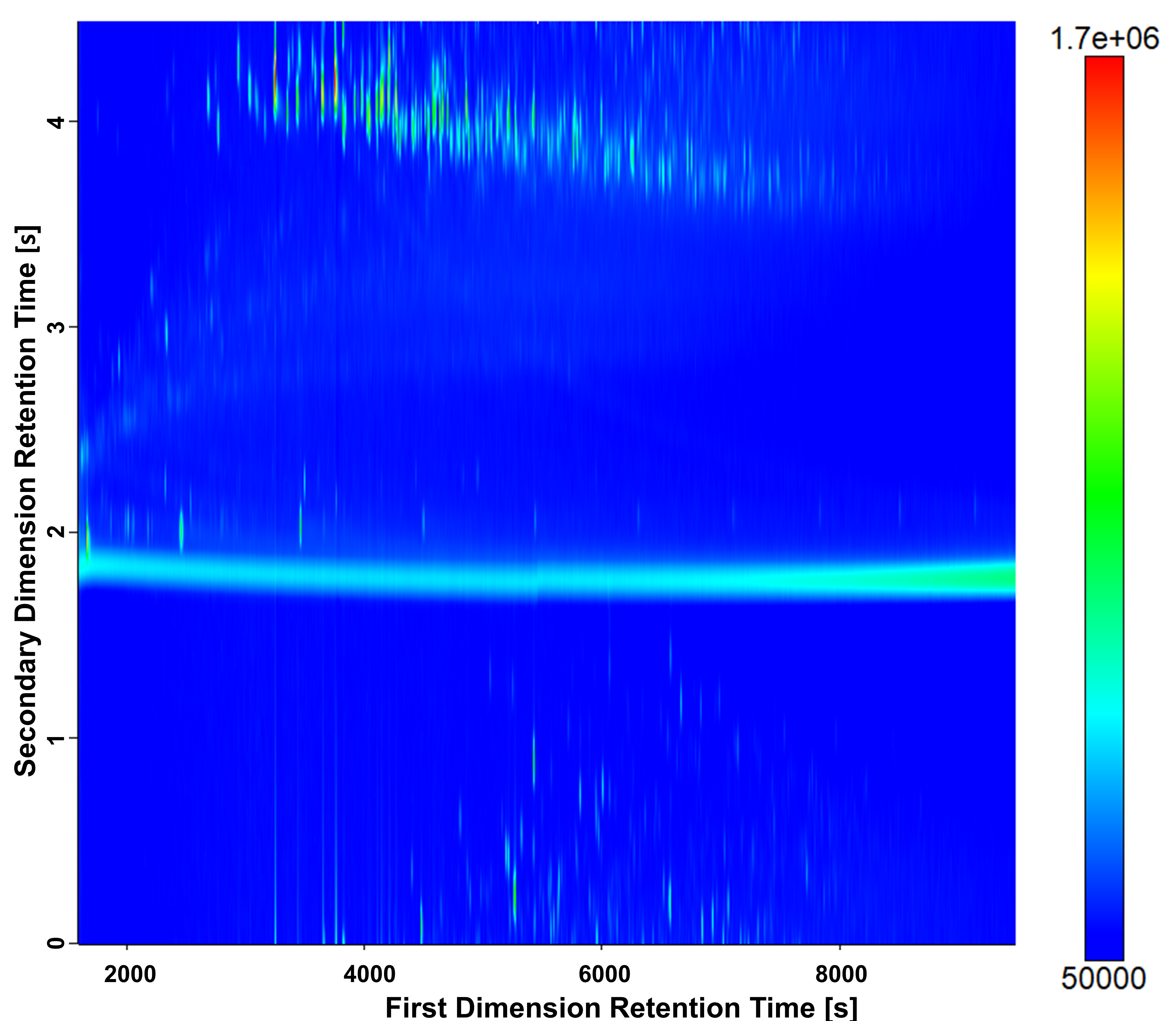

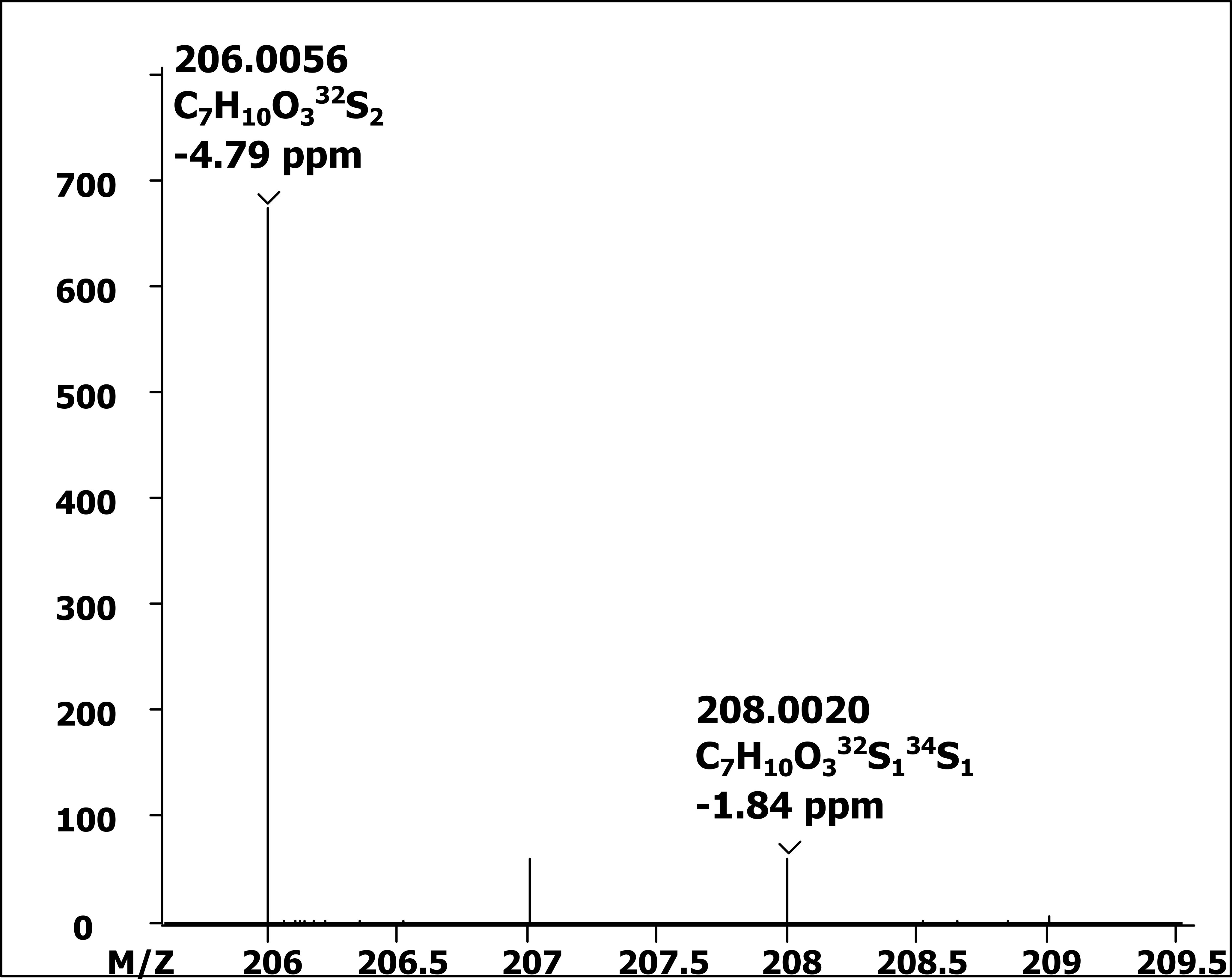


Fig S8 Isotopic Pattern of sulfur in the molecular ion [M]^+●^ (C_7_H_10_O_3_S_2_). Mass spectra of the most abundant peak in the SBS measurement (labeled in Fig 4) (zoomed 206-209.5 m/z). The two isotopic peaks of sulfur are labeled with their calculated m/z ratio, sum formula and mass error. Intensity of the second labeled peak (C_7_H_10_O_3_^32^S_1_^34^S_1_) is 8.8 % (674.6 to 59.8) of the peak from the molecular ion. The natural abundance of ^34^S is 4.4 % of ^32^S for one sulfur.

Table S1 Mass calibration parameters

| **Priority** | **Formula** | **Exact neutral mass** | **Expected m/z** |
| --- | --- | --- | --- |
| 1 | CF3 | 68.9952 | 68.9947 |
| Check Mass | C2F5 | 118.9920 | 118.9915 |
| 1 | C3F5 | 130.9920 | 130.9915 |
| 1 | C4F9 | 218.9856 | 218.9851 |
| 2 | C5F10N | 263.9871 | 263.9866 |
| Check Mass | C8F16N | 413.9775 | 413.9770 |
| 2 | C9F20N | 501.9711 | 501.9706 |

Table S2 Peak finding parameters of the ChromaTOF software

| **Parameter** |  |
| --- | --- |
| Start | Start of Run |
| End | End of Run |
| S/N | 10 |
| Peak Quality | 0.700 |
| Peak Confidence | 1.5 |
| Peak Width (s) | 0.2 |
| Intensity Threshold | 0.0 |

Table S3 Peak count of the refined precursor measurements including their proportion to the total detected peaks (S/N>100).

|  | **1** | **2** | **3** | **4** | **5** | **Average** | **SD** |
| --- | --- | --- | --- | --- | --- | --- | --- |
| CH DBE 0 | 110 | 102 | 106 | 94 | 104 | 103.2 | 5.9 |
| CH DBE 1 | 161 | 148 | 152 | 149 | 146 | 151.2 | 5.9 |
| CH DBE 2 | 162 | 162 | 183 | 168 | 168 | 168.6 | 8.6 |
| CH DBE 3 | 91 | 89 | 87 | 82 | 93 | 88.4 | 4.2 |
| CH DBE 4 | 98 | 95 | 95 | 89 | 95 | 94.4 | 3.3 |
| CH DBE 5 | 181 | 164 | 197 | 181 | 173 | 179.2 | 12.2 |
| CH DBE 6 | 170 | 155 | 189 | 173 | 164 | 170.2 | 12.6 |
| CH DBE 7 | 8 | 8 | 10 | 9 | 9 | 8.8 | 0.8 |
| CHS_1_ DBE 1 | 41 | 47 | 47 | 45 | 43 | 44.6 | 2.6 |
| CHS_1_ DBE 2 | 10 | 9 | 9 | 7 | 7 | 8.4 | 1.3 |
| CHS_1_ DBE 3 | 252 | 232 | 268 | 248 | 254 | 250.8 | 12.9 |
| CHS_1_ DBE 4 | 118 | 107 | 137 | 115 | 122 | 119.8 | 11.1 |
| CHS_1_ DBE 5 | 11 | 15 | 14 | 17 | 11 | 13.6 | 2.6 |
| CHS_1_ DBE 6 | 45 | 40 | 44 | 42 | 42 | 42.6 | 1.9 |
| CHS_1_ DBE 7 | 2 | 4 | 3 | 4 | 4 | 3.4 | 0.9 |
| CHS_2_ DBE 5 | 16 | 17 | 14 | 16 | 15 | 15.6 | 1.1 |
| CHS_2_ DBE 6 | 5 | 7 | 4 | 6 | 5 | 5.4 | 1.1 |
| CHO_1_ DBE 1 | 72 | 57 | 63 | 79 | 65 | 67.2 | 8.5 |
| CHO_1_ DBE 2 | 3 | 4 | 3 | 3 | 3 | 3.2 | 0.4 |
| CHO_1_ DBE 3 | 7 | 7 | 9 | 6 | 7 | 7.2 | 1.1 |
| CHO_1_ DBE 4 | 1 | 1 | 1 | 0 | 1 | 0.8 | 0.4 |
| CHN_1_ DBE 3 | 0 | 0 | 0 | 0 | 0 | 0.0 | 0.0 |
| CHN_1_ DBE 4 | 0 | 0 | 0 | 0 | 0 | 0.0 | 0.0 |
| Other | 386 | 360 | 343 | 312 | 383 | 356.8 | 30.6 |
| Overall | 1950 | 1830 | 1978 | 1845 | 1914 | 1903.4 | 64.5 |
| Classified [%] | 80.2 | 80.3 | 82.7 | 83.1 | 80.0 | 81.3 | 1.5 |

Table S4 Peak count of the distillate measurements including their proportion to the total detected peaks (S/N>100)

|  | **1** | **2** | **3** | **4** | **5** | **Average** | **SD** |
| --- | --- | --- | --- | --- | --- | --- | --- |
| CH DBE 0 | 115 | 99 | 113 | 91 | 99 | 103.4 | 10.2 |
| CH DBE 1 | 163 | 151 | 153 | 149 | 162 | 155.6 | 6.5 |
| CH DBE 2 | 162 | 180 | 179 | 169 | 165 | 171.0 | 8.2 |
| CH DBE 3 | 117 | 92 | 114 | 87 | 101 | 102.2 | 13.2 |
| CH DBE 4 | 110 | 95 | 114 | 86 | 98 | 100.6 | 11.4 |
| CH DBE 5 | 197 | 179 | 189 | 167 | 171 | 180.6 | 12.4 |
| CH DBE 6 | 185 | 168 | 182 | 156 | 164 | 171.0 | 12.2 |
| CH DBE 7 | 14 | 10 | 8 | 7 | 9 | 9.6 | 2.7 |
| CHS_1_ DBE 1 | 39 | 41 | 45 | 39 | 37 | 40.2 | 3.0 |
| CHS_1_ DBE 2 | 19 | 13 | 16 | 16 | 17 | 16.2 | 2.2 |
| CHS_1_ DBE 3 | 296 | 259 | 278 | 248 | 253 | 266.8 | 19.9 |
| CHS_1_ DBE 4 | 136 | 122 | 143 | 123 | 113 | 127.4 | 12.0 |
| CHS_1_ DBE 5 | 5 | 5 | 5 | 2 | 2 | 3.8 | 1.6 |
| CHS_1_ DBE 6 | 48 | 47 | 45 | 41 | 39 | 44.0 | 3.9 |
| CHS_1_ DBE 7 | 3 | 3 | 2 | 3 | 4 | 3.0 | 0.7 |
| CHS_2_ DBE 5 | 15 | 16 | 15 | 16 | 14 | 15.2 | 0.8 |
| CHS_2_ DBE 6 | 4 | 4 | 5 | 5 | 4 | 4.4 | 0.5 |
| CHO_1_ DBE 1 | 70 | 66 | 63 | 76 | 73 | 69.6 | 5.2 |
| CHO_1_ DBE 2 | 3 | 3 | 3 | 4 | 3 | 3.2 | 0.4 |
| CHO_1_ DBE 3 | 10 | 9 | 10 | 8 | 8 | 9.0 | 1.0 |
| CHO_1_ DBE 4 | 14 | 13 | 13 | 15 | 15 | 14.0 | 1.0 |
| CHN_1_ DBE 3 | 1 | 1 | 1 | 1 | 1 | 1.0 | 0.0 |
| CHN_1_ DBE 4 | 9 | 9 | 9 | 9 | 9 | 9.0 | 0.0 |
| Other | 540 | 392 | 515 | 363 | 395 | 441.0 | 80.4 |
| Overall | 2275 | 1977 | 2220 | 1881 | 1956 | 2061.8 | 174.3 |
| Classified [%] | 76.3 | 80.2 | 76.8 | 80.7 | 79.8 | 78.7 | 2.1 |

Table S5 Relative abundances in % of the replicate measurements of the refined precursor. Grubb’s tests were performed to examine for outliers. The *-labeled values highlight the outlier and their calculated G value (G>1.764 for n=5, α=0.01). The statistical values average, standard deviation (SD) and relative standard deviation (RSD) are calculated without outliers.

|  | **1** | **2** | **3** | **4** | **5** | **Grubbs test** | | **Average** | **SD** | **RSD** |
| --- | --- | --- | --- | --- | --- | --- | --- | --- | --- | --- |
|  |  |  |  |  |  | min | max |  |  |  |
| CH DBE 0 | 4.7E+00 | 4.7E+00 | 4.7E+00 | 4.9E+00 | 4.7E+00 | 0.75 | 1.75 | 4.7E+00 | 7.4E-02 | 1.6E-02 |
| CH DBE 1 | 8.9E+00 | 8.8E+00 | 9.1E+00 | 8.5E+00 | 8.6E+00 | 1.24 | 1.39 | 8.8E+00 | 2.3E-01 | 2.6E-02 |
| CH DBE 2 | 1.0E+01 | 1.1E+01 | 1.1E+01 | 1.1E+01 | 1.0E+01 | 0.72 | 1.64 | 1.1E+01 | 3.4E-01 | 3.2E-02 |
| CH DBE 3 | 2.6E+00 | 2.5E+00 | 2.7E+00 | 2.4E+00 | 2.5E+00 | 1.14 | 1.56 | 2.5E+00 | 1.3E-01 | 5.2E-02 |
| CH DBE 4 | 7.2E+00 | 7.2E+00 | 7.6E+00 | 7.0E+00 | 7.3E+00 | 1.21 | 1.45 | 7.3E+00 | 2.1E-01 | 2.9E-02 |
| CH DBE 5 | 3.7E+00 | 4.0E+00 | 3.6E+00 | 4.4E+00 | 3.7E+00 | 0.84 | 1.62 | 3.9E+00 | 3.1E-01 | 8.1E-02 |
| CH DBE 6 | 3.1E+00 | 3.2E+00 | 3.2E+00 | 3.9E+00* | 3.2E+00 | 0.61 | 1.78* | 3.2E+00 | 4.6E-02 | 1.4E-02 |
| CH DBE 7 | 3.4E-01 | 3.6E-01 | 3.7E-01 | 3.5E-01 | 4.2E-01 | 0.94 | 1.66 | 3.7E-01 | 3.0E-02 | 8.3E-02 |
| CHS_1_ DBE 1 | 1.7E+00 | 1.8E+00 | 1.7E+00 | 1.7E+00 | 2.0E+00 | 0.75 | 1.60 | 1.8E+00 | 1.1E-01 | 6.5E-02 |
| CHS_1_ DBE 2 | 1.4E-01 | 6.2E-02 | 1.3E-01 | 1.9E-01 | 9.9E-02 | 1.27 | 1.42 | 1.3E-01 | 4.9E-02 | 3.9E-01 |
| CHS_1_ DBE 3 | 2.6E+01 | 2.7E+01 | 2.7E+01 | 2.6E+01 | 2.7E+01 | 1.27 | 1.13 | 2.6E+01 | 5.2E-01 | 2.0E-02 |
| CHS_1_ DBE 4 | 6.0E+00 | 6.1E+00 | 6.6E+00 | 6.0E+00 | 6.0E+00 | 0.62 | 1.74 | 6.1E+00 | 2.9E-01 | 4.7E-02 |
| CHS_1_ DBE 5 | 4.3E-01 | 4.4E-01 | 4.4E-01 | 4.7E-01 | 4.1E-01 | 1.27 | 1.42 | 4.4E-01 | 2.2E-02 | 5.0E-02 |
| CHS_1_ DBE 6 | 2.7E+00 | 2.8E+00 | 2.8E+00 | 2.7E+00 | 2.7E+00 | 0.59 | 1.74 | 2.8E+00 | 4.3E-02 | 1.6E-02 |
| CHS_1_ DBE 7 | 1.3E-01 | 7.0E-01* | 9.0E-02 | 1.5E-01 | 1.5E-01 | 0.61 | 1.78* | 1.3E-01 | 2.9E-02 | 2.2E-01 |
| CHS_2_ DBE 5 | 9.0E-01 | 4.7E-01 | 1.1E+00 | 5.2E-01 | 5.1E-01 | 0.79 | 1.44 | 7.1E-01 | 3.0E-01 | 4.2E-01 |
| CHS_2_ DBE 6 | 1.9E-01 | 2.1E-01 | 1.8E-01 | 1.8E-01 | 1.9E-01 | 0.82 | 1.69 | 1.9E-01 | 1.2E-02 | 6.4E-02 |
| CHO_1_ DBE 1 | 8.2E-01 | 8.3E-01 | 8.0E-01 | 7.5E-01 | 7.4E-01 | 1.21 | 1.05 | 7.9E-01 | 4.2E-02 | 5.3E-02 |
| CHO_1_ DBE 2 | 6.6E-02 | 7.0E-02 | 6.8E-02 | 6.6E-02 | 6.9E-02 | 1.03 | 1.11 | 6.8E-02 | 1.8E-03 | 2.6E-02 |
| CHO_1_ DBE 3 | 5.4E-02 | 4.8E-02 | 5.6E-02 | 4.1E-02 | 4.9E-02 | 1.45 | 1.14 | 4.9E-02 | 6.1E-03 | 1.2E-01 |
| CHO_1_ DBE 4 | 4.1E-03 | 3.1E-03 | 3.2E-03 | N.D. | 2.4E-02* | 0.71 | 1.77* | 2.6E-03 | 1.8E-03 | 6.9E-01 |
| CHN_1_ DBE 3 | N.D. | N.D. | N.D. | N.D. | N.D. | N.A. | N.A. | N.A. | N.A. | N.A. |
| CHN_1_ DBE 4 | N.D. | N.D. | N.D. | N.D. | N.D. | N.A. | N.A. | N.A. | N.A. | N.A. |
| Overall | 8.0E+01 | 8.2E+01 | 8.4E+01 | 8.1E+01 | 8.1E+01 | 0.98 | 1.58 | 8.1E+01 | 1.5E+00 | 1.8E-02 |

Table S6 Relative abundances in % of the replicate measurements of the distillate Grubb’s tests were performed to examine for outliers. The *-labeled values highlight the outlier and their calculated G value (G>1.764 for n=5, α=0.01). The statistical values average, standard deviation (SD) and relative standard deviation (RSD) are calculated without outliers.

|  | **1** | **2** | **3** | **4** | **5** | **Grubbs test** | | **Average** | **SD** | **RSD** |
| --- | --- | --- | --- | --- | --- | --- | --- | --- | --- | --- |
|  |  |  |  |  |  | min | max |  |  |  |
| CH DBE 0 | 4.3E+00 | 4.3E+00 | 4.0E+00 | 4.4E+00 | 4.4E+00 | 1.73 | 0.71 | 4.3E+00 | 1.6E-01 | 3.8E-02 |
| CH DBE 1 | 7.5E+00 | 8.2E+00 | 7.3E+00 | 8.3E+00 | 8.8E+00 | 1.14 | 1.25 | 8.0E+00 | 6.4E-01 | 8.0E-02 |
| CH DBE 2 | 1.0E+01 | 1.1E+01 | 1.0E+01 | 1.0E+01 | 1.1E+01 | 0.91 | 1.23 | 1.0E+01 | 3.9E-01 | 3.7E-02 |
| CH DBE 3 | 4.0E+00 | 3.7E+00 | 3.4E+00 | 3.7E+00 | 3.9E+00 | 1.45 | 1.17 | 3.7E+00 | 2.4E-01 | 6.5E-02 |
| CH DBE 4 | 6.8E+00 | 6.9E+00 | 6.7E+00 | 6.9E+00 | 6.9E+00 | 1.63 | 0.72 | 6.9E+00 | 9.4E-02 | 1.4E-02 |
| CH DBE 5 | 3.4E+00 | 3.2E+00 | 3.3E+00 | 3.5E+00 | 3.2E+00 | 0.97 | 1.20 | 3.3E+00 | 1.0E-01 | 3.1E-02 |
| CH DBE 6 | 3.0E+00 | 2.6E+00 | 2.9E+00 | 3.0E+00 | 2.8E+00 | 1.49 | 0.93 | 2.9E+00 | 1.8E-01 | 6.1E-02 |
| CH DBE 7 | 4.0E-01 | 3.4E-01 | 3.2E-01 | 3.4E-01 | 3.4E-01 | 0.95 | 1.70 | 3.5E-01 | 3.3E-02 | 9.5E-02 |
| CHS_1_ DBE 1 | 1.7E+00 | 1.6E+00 | 1.8E+00 | 1.4E+00 | 1.7E+00 | 1.58 | 1.08 | 1.7E+00 | 1.5E-01 | 9.0E-02 |
| CHS_1_ DBE 2 | 3.6E-01 | 3.7E-01 | 3.7E-01 | 4.0E-01 | 4.1E-01 | 1.09 | 1.29 | 3.8E-01 | 2.3E-02 | 6.1E-02 |
| CHS_1_ DBE 3 | 2.5E+01 | 2.5E+01 | 2.5E+01 | 2.6E+01 | 2.6E+01 | 1.13 | 0.96 | 2.5E+01 | 5.0E-01 | 2.0E-02 |
| CHS_1_ DBE 4 | 6.2E+00 | 6.2E+00 | 6.2E+00 | 6.4E+00 | 6.4E+00 | 1.06 | 1.26 | 6.3E+00 | 1.2E-01 | 1.9E-02 |
| CHS_1_ DBE 5 | 9.2E-02 | 1.1E-01 | 9.4E-02 | 8.1E-02 | 8.2E-02 | 0.95 | 1.52 | 9.1E-02 | 1.1E-02 | 1.2E-01 |
| CHS_1_ DBE 6 | 2.5E+00 | 2.7E+00 | 2.5E+00 | 2.7E+00 | 2.6E+00 | 1.04 | 1.04 | 2.6E+00 | 1.0E-01 | 3.9E-02 |
| CHS_1_ DBE 7 | 1.3E-01 | 1.4E-01 | 7.6E-02 | 1.4E-01 | 1.4E-01 | 1.75 | 0.61 | 1.3E-01 | 2.8E-02 | 2.3E-01 |
| CHS_2_ DBE 5 | 2.3E+00 | 3.5E+00 | 4.6E-01 | 9.5E-01 | 2.3E+00 | 1.20 | 1.32 | 1.9E+00 | 1.2E+00 | 6.3E-01 |
| CHS_2_ DBE 6 | 2.6E+00 | 1.2E+00 | 1.7E-01 | 1.8E-01 | 1.7E-01 | 0.66 | 1.61 | 8.6E-01 | 1.1E+00 | 1.2E+00 |
| CHO_1_ DBE 1 | 8.0E-01 | 7.6E-01 | 7.9E-01 | 7.9E-01 | 7.6E-01 | 1.02 | 1.21 | 7.8E-01 | 1.7E-02 | 2.2E-02 |
| CHO_1_ DBE 2 | 7.1E-02 | 7.8E-02 | 7.0E-02 | 8.3E-02 | 8.1E-02 | 1.13 | 1.09 | 7.7E-02 | 5.9E-03 | 7.6E-02 |
| CHO_1_ DBE 3 | 9.5E-02 | 1.0E-01 | 9.7E-02 | 1.0E-01 | 1.0E-01 | 1.38 | 0.92 | 9.9E-02 | 3.0E-03 | 3.1E-02 |
| CHO_1_ DBE 4 | 3.4E-01 | 3.8E-01 | 2.8E-01 | 3.2E-01 | 3.6E-01 | 1.46 | 1.17 | 3.4E-01 | 3.9E-02 | 1.2E-01 |
| CHN_1_ DBE 3 | 2.0E-03 | 2.0E-03 | 1.0E-03* | 2.0E-03 | 2.0E-03 | 1.79* | 0.45 | 1.8E-03 | 4.5E-04 | 2.5E-01 |
| CHN_1_ DBE 4 | 5.0E-02 | 4.8E-02 | 4.7E-02 | 1.4E-01* | 5.5E-02 | 0.52 | 1.78* | 5.0E-02 | 3.6E-03 | 7.1E-02 |
| Overall | 8.1E+01 | 8.3E+01 | 7.6E+01 | 8.0E+01 | 8.2E+01 | 1.63 | 0.89 | 4.3E+00 | 1.6E-01 | 3.8E-02 |

Table S7 Relative abundances of the replicate measurements of SBS and the calculated values average, standard deviation (SD) and relative standard deviation (RSD)

|  | **DBE** | **1** | **2** | **3** | **Average** | **SD** | **RSD** |
| --- | --- | --- | --- | --- | --- | --- | --- |
| CHO_3_S_1_ |  | | | | | | |
|  | 4 | 20.4% | 21.2% | 20.4% | 20.7% | 0.4% | 2.0% |
|  | 5 | 2.0% | 1.7% | 2.0% | 1.9% | 0.2% | 8.8% |
|  | 7 | 0.3% | 0.3% | 0.3% | 0.3% | <0.1% | 0.8% |
| CHO_3_S_2_ |  | | | | | | |
|  | 3 | 58.2% | 58.3% | 58.2% | 58.2% | 0.1% | 0.2% |
|  | 4 | 3.0% | 2.6% | 3.0% | 2.8% | 0.3% | 8.9% |
|  | 6 | 1.6% | 1.5% | 1.6% | 1.6% | <0.1% | 0.9% |
|  | 7 | <0.1% | <0.1% | <0.1% | <0.1% | <0.1% | 4.9% |
| CHO_3_S_3_ |  | | | | | | |
|  | 3 | N.D. | N.D. | N.D. | N.A. | N.A. | N.A. |
|  | 4 | N.D. | N.D. | N.D. | N.A. | N.A. | N.A. |
|  | 6 | 0.1% | <0.1% | 0.1% | 0.1% | <0.1% | 30.2% |
| Non-sulfonated | 3-6 | 4.9% | 4.2% | 5.4% | 4.8% | 0.6% | 12.3% |

Table S8 Peak count of the SBS measurements including their proportion to the total detected peaks (S/N>100)

|  | **1** | **2** | **3** | **Average** | **SD** |
| --- | --- | --- | --- | --- | --- |
| C7_ CHO3S1_4 | 1 | 1 | 1 | 1.0 | 0.0 |
| C8_ CHO3S1_4 | 3 | 3 | 3 | 3.0 | 0.0 |
| C9_ CHO3S1_4 | 9 | 9 | 9 | 9.0 | 0.0 |
| C10_ CHO3S1_4 | 14 | 19 | 14 | 15.7 | 2.9 |
| C11_ CHO3S1_4 | 21 | 22 | 21 | 21.3 | 0.6 |
| C12_ CHO3S1_4 | 8 | 10 | 8 | 8.7 | 1.2 |
| C13_ CHO3S1_4 | 4 | 3 | 4 | 3.7 | 0.6 |
| C14_ CHO3S1_4 | 3 | 6 | 3 | 4.0 | 1.7 |
| C15_ CHO3S1_4 | 2 | 2 | 2 | 2.0 | 0.0 |
| C16_ CHO3S1_4 | 0 | 1 | 0 | 0.3 | 0.6 |
| C10_ CHO3S1_5 | 2 | 2 | 2 | 2.0 | 0.0 |
| C11_ CHO3S1_5 | 7 | 8 | 7 | 7.3 | 0.6 |
| C12_ CHO3S1_5 | 9 | 10 | 9 | 9.3 | 0.6 |
| C13_ CHO3S1_5 | 3 | 4 | 3 | 3.3 | 0.6 |
| C14_ CHO3S1_5 | 1 | 1 | 1 | 1.0 | 0.0 |
| C11_ CHO3S1_7 | 2 | 2 | 2 | 2.0 | 0.0 |
| C12_ CHO3S1_7 | 4 | 3 | 4 | 3.7 | 0.6 |
| C13_ CHO3S1_7 | 3 | 3 | 3 | 3.0 | 0.0 |
| C5_ CHO3S2_3 | 1 | 1 | 1 | 1.0 | 0.0 |
| C6_ CHO3S2_3 | 4 | 5 | 4 | 4.3 | 0.6 |
| C7_ CHO3S2_3 | 8 | 9 | 8 | 8.3 | 0.6 |
| C8_ CHO3S2_3 | 9 | 9 | 9 | 9.0 | 0.0 |
| C9_ CHO3S2_3 | 8 | 10 | 8 | 8.7 | 1.2 |
| C10_ CHO3S2_3 | 19 | 22 | 19 | 20.0 | 1.7 |
| C11_ CHO3S2_3 | 31 | 33 | 31 | 31.7 | 1.2 |
| C12_ CHO3S2_3 | 28 | 32 | 28 | 29.3 | 2.3 |
| C13_ CHO3S2_3 | 15 | 19 | 15 | 16.3 | 2.3 |
| C14_ CHO3S2_3 | 15 | 19 | 15 | 16.3 | 2.3 |
| C15_ CHO3S2_3 | 15 | 16 | 15 | 15.3 | 0.6 |
| C16_ CHO3S2_3 | 7 | 10 | 7 | 8.0 | 1.7 |
| C17_ CHO3S2_3 | 6 | 5 | 6 | 5.7 | 0.6 |
| C18_ CHO3S2_3 | 3 | 4 | 3 | 3.3 | 0.6 |
| C19_ CHO3S2_3 | 0 | 1 | 0 | 0.3 | 0.6 |
| C8_ CHO3S2_4 | 1 | 1 | 1 | 1.0 | 0.0 |
| C9_ CHO3S2_4 | 3 | 4 | 3 | 3.3 | 0.6 |
| C10_ CHO3S2_4 | 2 | 1 | 2 | 1.7 | 0.6 |
| C11_ CHO3S2_4 | 4 | 5 | 4 | 4.3 | 0.6 |
| C12_ CHO3S2_4 | 2 | 4 | 2 | 2.7 | 1.2 |
| C13_ CHO3S2_4 | 4 | 6 | 4 | 4.7 | 1.2 |
| C14_ CHO3S2_4 | 9 | 8 | 9 | 8.7 | 0.6 |
| C15_ CHO3S2_4 | 2 | 2 | 2 | 2.0 | 0.0 |
| C16_ CHO3S2_4 | 1 | 1 | 1 | 1.0 | 0.0 |
| C17_ CHO3S2_4 | 0 | 1 | 0 | 0.3 | 0.6 |
| C18_ CHO3S2_4 | 1 | 1 | 1 | 1.0 | 0.0 |
| C9_ CHO3S2_6 | 1 | 1 | 1 | 1.0 | 0.0 |
| C10_ CHO3S2_6 | 2 | 2 | 2 | 2.0 | 0.0 |
| C11_ CHO3S2_6 | 8 | 8 | 8 | 8.0 | 0.0 |
| C12_ CHO3S2_6 | 7 | 6 | 7 | 6.7 | 0.6 |
| C12_ CHO3S2_7 | 1 | 1 | 1 | 1.0 | 0.0 |
| C14_ CHO3S3_6 | 2 | 1 | 2 | 1.7 | 0.6 |
| Over all peaks | 436 | 513 | 436 | 461.7 | 44.5 |
| Classified peaks | 72.2% | 69.6% | 72.2% | 71.4% | 1.5% |

Table S9 Relative abundance of the three repetitions of the SBS measurement. The carbon number, elemental composition and DBE value separate the classes (merge by underscores)

|  | **1** | **2** | **3** | **Average** | **SD** | **RSD** |
| --- | --- | --- | --- | --- | --- | --- |
| C7_CHO3S1_4 | 0.1% | 0.1% | 0.1% | 0.1% | <0.1% | 5.8% |
| C8_ CHO3S1_4 | 1.9% | 2.1% | 1.9% | 2.0% | 0.1% | 4.2% |
| C9_ CHO3S1_4 | 6.4% | 7.2% | 6.4% | 6.7% | 0.5% | 7.2% |
| C10_ CHO3S1_4 | 5.9% | 5.3% | 5.9% | 5.7% | 0.4% | 6.4% |
| C11_ CHO3S1_4 | 4.6% | 4.9% | 4.6% | 4.7% | 0.2% | 3.6% |
| C12_ CHO3S1_4 | 0.8% | 1.0% | 0.8% | 0.8% | 0.2% | 18.7% |
| C13_ CHO3S1_4 | 0.3% | 0.2% | 0.3% | 0.3% | 0.1% | 17.9% |
| C14_ CHO3S1_4 | 0.3% | 0.2% | 0.3% | 0.2% | 0.1% | 32.6% |
| C15_ CHO3S1_4 | 0.1% | 0.1% | 0.1% | 0.1% | <0.1% | 29.9% |
| C16_ CHO3S1_4 | <0.1% | 0.1% | <0.1% | <0.1% | <0.1% | 173.2% |
| C10_ CHO3S1_5 | 0.2% | 0.2% | 0.2% | 0.2% | <0.1% | 4.7% |
| C11_ CHO3S1_5 | 0.8% | 0.7% | 0.8% | 0.7% | 0.1% | 7.2% |
| C12_ CHO3S1_5 | 0.7% | 0.6% | 0.7% | 0.7% | 0.1% | 11.9% |
| C13_ CHO3S1_5 | 0.2% | 0.2% | 0.2% | 0.2% | <0.1% | 10.6% |
| C14_ CHO3S1_5 | <0.1% | <0.1% | <0.1% | <0.1% | <0.1% | 1.4% |
| C11_ CHO3S1_7 | 0.1% | 0.1% | 0.1% | 0.1% | <0.1% | 21.0% |
| C12_ CHO3S1_7 | 0.1% | 0.2% | 0.1% | 0.2% | <0.1% | 7.0% |
| C13_ CHO3S1_7 | 0.1% | 0.1% | 0.1% | 0.1% | <0.1% | 12.4% |
| C5_ CHO3S2_3 | <0.1% | 0.1% | <0.1% | 0.1% | <0.1% | 7.9% |
| C6_ CHO3S2_3 | 1.1% | 1.2% | 1.1% | 1.1% | 0.1% | 8.3% |
| C7_ CHO3S2_3 | 7.4% | 8.0% | 7.4% | 7.6% | 0.3% | 3.9% |
| C8_ CHO3S2_3 | 11.4% | 12.1% | 11.4% | 11.6% | 0.4% | 3.8% |
| C9_ CHO3S2_3 | 9.4% | 9.7% | 9.4% | 9.5% | 0.2% | 2.0% |
| C10_ CHO3S2_3 | 7.9% | 6.3% | 7.9% | 7.4% | 0.9% | 12.3% |
| C11_ CHO3S2_3 | 7.2% | 7.6% | 7.2% | 7.3% | 0.3% | 3.6% |
| C12_ CHO3S2_3 | 4.3% | 3.5% | 4.3% | 4.0% | 0.5% | 11.3% |
| C13_ CHO3S2_3 | 2.4% | 2.9% | 1.8% | 2.3% | 0.6% | 23.9% |
| C14_ CHO3S2_3 | 2.9% | 3.0% | 3.3% | 3.1% | 0.2% | 6.4% |
| C15_ CHO3S2_3 | 2.5% | 2.8% | 2.7% | 2.6% | 0.1% | 5.4% |
| C16_ CHO3S2_3 | 0.9% | 0.8% | 0.9% | 0.8% | <0.1% | 5.9% |
| C17_ CHO3S2_3 | 0.7% | 0.4% | 0.7% | 0.6% | 0.2% | 29.2% |
| C18_ CHO3S2_3 | 0.2% | <0.1% | 0.2% | 0.1% | 0.1% | 86.6% |
| C8_ CHO3S2_4 | <0.1% | 0.1% | <0.1% | <0.1% | <0.1% | 108.6% |
| C9_ CHO3S2_4 | 0.2% | 0.2% | 0.2% | 0.2% | <0.1% | 4.8% |
| C10_ CHO3S2_4 | 0.1% | 0.1% | 0.1% | 0.1% | <0.1% | 22.3% |
| C11_ CHO3S2_4 | 0.2% | 0.2% | 0.2% | 0.2% | <0.1% | 17.4% |
| C12_ CHO3S2_4 | 0.3% | 0.3% | 0.3% | 0.3% | <0.1% | 0.8% |
| C13_ CHO3S2_4 | 0.7% | 0.7% | 0.7% | 0.7% | <0.1% | 5.4% |
| C14_ CHO3S2_4 | 1.0% | 0.8% | 1.0% | 0.9% | 0.1% | 11.1% |
| C15_ CHO3S2_4 | 0.2% | 0.2% | 0.2% | 0.2% | 0.2% | <0.1% |
| C16_ CHO3S2_4 | 0.1% | 0.1% | 0.1% | 0.1% | <0.1% | 7.1% |
| C18_ CHO3S2_4 | 0.1% | <0.1% | 0.1% | 0.1% | 0.1% | 86.6% |
| C9_ CHO3S2_6 | <0.1% | <0.1% | <0.1% | <0.1% | <0.1% | 4.6% |
| C10_ CHO3S2_6 | 0.5% | 0.5% | 0.5% | 0.5% | <0.1% | 4.5% |
| C11_ CHO3S2_6 | 0.8% | 0.8% | 0.8% | 0.8% | <0.1% | 2.6% |
| C12_ CHO3S2_6 | 0.3% | 0.2% | 0.3% | 0.3% | <0.1% | 7.3% |
| C12_ CHO3S2_7 | <0.1% | <0.1% | <0.1% | <0.1% | <0.1% | 4.9% |
| C14_ CHO3S3_6 | 0.1% | <0.1% | 0.1% | 0.1% | <0.1% | 30.2% |
| Overall ion current | 85.6% | 85.7% | 85.6% | 85.6% | 0.1% | 0.1% |
